# Multipotent Poly(Tertiary Amine‐Oxide) Micelles for Efficient Cancer Drug Delivery

**DOI:** 10.1002/advs.202200173

**Published:** 2022-02-20

**Authors:** Jiajia Xiang, Yihuai Shen, Yifan Zhang, Xin Liu, Quan Zhou, Zhuxian Zhou, Jianbin Tang, Shiqun Shao, Youqing Shen

**Affiliations:** ^1^ Zhejiang Key Laboratory of Smart BioMaterials and Center for Bionanoengineering College of Chemical and Biological Engineering Zhejiang University Hangzhou 310027 China; ^2^ ZJU‐Hangzhou Global Scientific and Technological Innovation Center Hangzhou 311215 China; ^3^ Key Laboratory of Biomass Chemical Engineering of the Ministry of Education College of Chemical and Biological Engineering, Hangzhou Zhejiang University Hangzhou 310027 China; ^4^ Department of Orthopaedic Surgery Sir Run Run Shaw Hospital Medical College of Zhejiang University Hangzhou 310016 China; ^5^ School of Basic Medical Sciences Zhejiang University Hangzhou 310058 China

**Keywords:** cell membrane affinity, micelle, mitochondria targeting, poly(tertiary amine‐oxide), transcytosis

## Abstract

The cancer drug delivery process involves a series of biological barriers, which require the nanomedicine to exhibit different, even opposite properties for high therapeutic efficacy. The prevailing design philosophy, i.e., integrating these properties within one nanomedicine via on‐demand property transitions such as PEGylation/dePEGylation, complicates nanomedicines’ composition and thus impedes clinical translation. Here, polyzwitterionic micelles of poly(tertiary amine‐oxide)‐*block*‐poly(*ε*‐caprolactone) (PTAO‐PCL) amphiphiles that enable all the required functions are presented. The zwitterionic nature and unique cell membrane affinity confer the PTAO micelles long blood circulation, efficient tumor accumulation and penetration, and fast cellular internalization. The mitochondrial targeting capability allows drug delivery into the mitochondria to induce mitochondrial dysfunction and overcome tumor multidrug resistance. As a result, the PTAO/drug micelles exhibit potent anticancer efficacy. This simple yet multipotent carrier system holds great promise as a generic platform for potential clinical translation.

## Graphical abstract

Polyzwitterionic micelles of poly(tertiary amine‐oxide)‐*block*‐poly(*ε*‐caprolactone) amphiphiles have been developed as a generic platform for efficient cancer drug delivery. The micelles' unique affinity toward cell membrane enables long blood circulation through red blood cell‐hitchhiking, transcytosis‐mediated enhanced tumor accumulation and penetration, fast cellular internalization, and finally mitochondrial targeting to overcome tumor multidrug resistance.

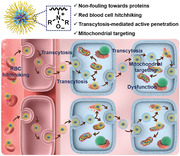

## 1 Introduction

Chemotherapy is an effective way to treat many cancers but can be confounded by severe adverse effects and drug resistance.^[^ ^1^ ^]^ Nanomedicine is one of the promising strategies that may address these issues.^[^ ^2^ ^]^ Of particular interest are those formulated in polymeric micelles. Typical polymeric micelles are self‐assembled from amphiphilic polymers and feature core‐shell structures.^[^ ^3^ ^]^ These structural characteristics enable good solubilization of hydrophobic agents encapsulated in the lipophilic core and long blood circulation time provided by the hydrophilic shell.^[^ ^4^ ^]^ Polymeric micelles also hold advantages in easy preparation and high scale‐up feasibility.^[^ ^5^ ^]^ Notably, a poly(ethylene glycol)‐*block*‐poly(lactide) micelle formulation of paclitaxel (Genexol‐PM) has been approved for clinical use.^[^ ^6^ ^]^ While the micellar formulation can mitigate many adverse effects, it fails to enhance the therapeutic efficacy.^[^ ^7^ ^]^

To sufficiently elicit the therapeutic activity, an ideal micellar nanomedicine should be capable of long blood circulation, specific tumor accumulation, deep tumor penetration, readily cellular internalization, precise subcellular localization if applicable, and efficient drug release.^[^ ^8^ ^]^ However, the properties required to achieve these capacities are generally conflicting. Taking commonly used poly(ethylene glycol)‐poly(*ε*‐caprolactone) (PEG‐PCL) micelles as an instance, while the stealthy PEG coating facilitates long blood circulation, it compromises cellular internalization. Rational design to enable spontaneous property transitions in response to various endogenous and/or exogenous stimuli at specific delivery nodes is a common strategy to tackle these dilemmas.^[^ ^9^ ^]^ For example, the introduction of a tumor‐acid‐labile linker between the PEG chain and the hydrophobic segment could achieve dePEGylation in the tumor microenvironment, resolving the PEG dilemma.^[^ ^9b^ ^]^ However, such all‐into‐one strategies may complicate the composition of nanomedicines and decrease the clinical translation potential.^[^ ^10^ ^]^ Moreover, the heterogeneous and dynamic nature of tumors may disable the designed micelles to achieve the supposed property transitions.^[^ ^11^ ^]^

We have recently reported a phospholipid‐affinitive poly(tertiary amine oxide) (PTAO), poly(2‐(*N*‐oxide‐*N*,*N*‐diethylamino)ethyl methacrylate) (OPDEA). The polyzwitteronic nature renders OPDEA non‐fouling toward proteins, enabling long blood circulation. The phospholipid affinity allows OPDEA to weakly bind to cells, which can trigger transcytosis‐mediated active extravasation and tumor penetration, as well as efficient cellular internalization.^[^ ^12^ ^]^ Compared with passive diffusion‐based extravasation and penetration that relies on the leaky tumor vasculatures and is generally limited by the dense and heterogeneous tumor microenvironment, transcytosis‐mediated processes leverage the intrinsic active transport pathways of endothelial cells and tumor cells and can significantly improve the tumor accumulation and infiltration of nanomedicines.^[^ ^13^ ^]^ OPDEA also exhibits a remarkable mitochondrial targeting ability.^[^ ^14^ ^]^ Mitochondria produce adenosine triphosphate (ATP) for ATP‐binding cassette transporters such as P‐glycoprotein to function as drug efflux pumps^[^ ^15^ ^]^ and may have mitochondrial DNA mutations conferring drug resistance.^[^ ^16^ ^]^ Thus, targeting mitochondria to disrupt ATP synthesis and induce DNA injury can be a straightforward way to overcome multidrug resistance (MDR) of tumor cells.

**Scheme 1:**
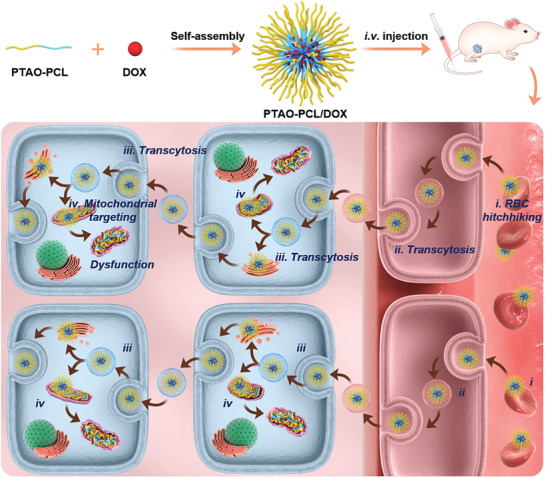
Schematic illustration of PTAO‐PCL micelles for cancer drug delivery. a) The encapsulation of DOX into PTAO micelles self‐assembled from PTAO‐PCL block copolymers. b) After intravenous injection, PTAO micelles (i) circulate long in blood via red blood cell (RBC) hitchhiking and (ii) attach on cell membranes to trigger transcytosis‐mediated extravasation and (iii) subsequent active tumor penetration. Inside tumor cells, a portion of PTAO micelles (iv) target mitochondria and induce cell death.

Prompted by these distinguished properties of OPDEA, we propose here to develop PTAO micelles as a drug delivery platform, which may prolong the blood circulation time, increase drug accumulation in tumors, promote tumor penetration and cellular internalization, and even overcome the MDR of tumor cells, given the critical role of mitochondria involving MDR,^[^ ^17^ ^]^ ultimately achieving significant therapeutic outcomes. For proof of concept, we chose PCL as the hydrophobic segment and constructed two kinds of PTAO‐PCL block copolymer (OPDMA‐PCL and OPDEA‐PCL) as carriers for doxorubicin (DOX) delivery (**Scheme** **1**). The DOX‐loaded micelles could reverse the MDR of MCF‐7/ADR tumors and achieve enhanced anticancer efficacy.

## 2 Results and Discussion

### 2.1 Preparation and Characterization of DOX‐Loaded PTAO‐PCL Micelles

**Figure 1:**
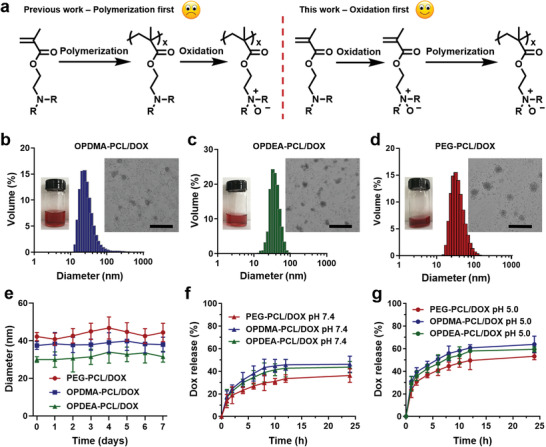
Characterization of DOX‐loaded micelles. a) Strategies for PTAO synthesis. b–d) Size distribution and TEM images of b) OPDMA‐PCL/DOX, c) OPDEA‐PCL/DOX, and d) PEG‐PCL/DOX micelles. Scale bar: 200 µm. e) The size variation of the DOX‐loaded micelles in full culture medium over 1 week. f, g) The drug release profiles of the DOX‐loaded micelles at f) pH 7.4 and g) pH 5.0.

OPDMA‐PCL and OPDEA‐PCL were synthesized as shown in Scheme S1 (Supporting Information). We used to prepare PTAO through post‐oxidation of poly(tertiary amine) using hydrogen peroxide^[^ ^14^ ^]^ or meta‐chloroperbenzoic acid (mCPBA),^[^ ^12^ ^]^ which requires strict control of reaction conditions. In this work, we developed a more facile method to produce PTAO‐containing block copolymers with defined structures (**Figure** **1**a). TAO monomers (ODMA and ODEA) were first produced by oxidizing *N*,*N*‐dimethylaminoethyl methacrylate (DMA) and *N*,*N*‐diethylaminoethyl methacrylate (DEA). mCPBA was selected as the oxidizing agent, as its activity does not affect the double bond of methacrylate.^[^ ^18^ ^]^ After column purification, ODMA and ODEA were obtained as colorless crystals. The chemical structures of these two monomers were confirmed by ^1^H‐NMR spectra (Figures S1 and S2, Supporting Information). Next, heterobifunctional PCL was synthesized via ring‐opening polymerization of *ε*‐caprolactone using 5‐(Boc‐amino)‐1‐pentanol as the initiator and diphenyl phosphate as the catalyst (Figure S3, Supporting Information), which then reacted with 2‐bromoisobutyryl bromide to yield PCL‐Br (Figure S4, Supporting Information). Subsequently, atom transfer radical polymerization^[^ ^19^ ^]^ of ODMA and ODEA was performed to produce OPDMA‐PCL and OPDEA‐PCL copolymers with PCL‐Br as the initiator. Each block of the copolymers was controlled to be ≈5 kDa (Figures S5 and S6, Supporting Information). Meanwhile, a PEG‐PCL block copolymer was synthesized as the control (Figure S7, Supporting Information). All the polymers were labeled with 1.0 wt.% of Cyanine 5 (Cy5) to afford fluorescently labeled carriers (OPDMA‐^Cy5^PCL, OPDEA‐^Cy5^PCL_,_ and PEG‐^Cy5^PCL; Scheme S2, Supporting Information).

The OPDMA‐PCL and OPDEA‐PCL copolymers could self‐assemble into well‐defined micelles in aqueous solutions. The respective diameters were 29 and 25 nm, and the zeta potentials were −2.08 and −3.46 mV (Table S1, Supporting Information). For comparison, the PEG‐PCL micelles had an average size of 31 nm and zeta potential of −2.85 mV. Encapsulation of DOX into these micelles was achieved through a nanoprecipitation method. The average sizes of the OPDMA‐PCL/DOX, OPDEA‐PCL/DOX, and PEG‐PCL/DOX micelles were 34.5, 27.7, and 37.4 nm, respectively, as determined by dynamic laser scattering (DLS) and transmission electron microscope (TEM; Figure 1b–d). Notably, the DOX‐loaded PTAO micelles were slightly negatively charged (Table S1, Supporting Information), which might benefit the stealth of the nanomedicines in blood circulation. The drug loading contents of OPDMA‐PCL/DOX and OPDEA‐PCL/DOX were 15.7% and 14.9%, respectively, with the corresponding loading efficiency of 82.3% and 80.8%, comparable to that of PEG‐PCL/DOX (16.3%; 85.4%; Table S1, Supporting Information). OPDMA‐PCL and OPDEA‐PCL also had a similar critical micelle concentration (CMC) of ≈20 µg mL^−1^, lower than that of PEG‐PCL (Figure S8, Supporting Information). Due to the polyzwitterionic corona and the low CMC values, the OPDMA‐PCL/DOX and OPDMA‐PCL/DOX micelles could keep stable in phosphate‐buffered saline (PBS) or full culture medium for one week, with no significant change in particle size (Figure 1e; Figure S9, Supporting Information).

The drug release kinetics of OPDMA‐PCL/DOX, OPDEA‐PCL/DOX, and PEG‐PCL/DOX was investigated in PBS at pH 7.4 and 5.0 to mimic different physiological environments. All the micelles showed sustained release profiles. At pH 7.4, the DOX release ratios of OPDMA‐PCL/DOX, OPDEA‐PCL/DOX, and PEG‐PCL/DOX after 24 h incubation were 46.2%, 43.6%, and 36.2%, respectively (Figure 1f). Acidic pH accelerated DOX release from all the formulations (Figure 1g) due to the protonation of DOX at acidic pH, which greatly increased its water solubility and rendered it squeezed out from the hydrophobic micelle cores.^[^ ^20^ ^]^ Once the DOX concentration in the medium is close to that inside the micelles, the concentration difference‐driven drug release will be slowed down, resulting in no complete release. Notably, the drug release rates of PTAO‐PCL/DOX were slightly fast than PEG‐PCL/DOX, possibly due to the more hydrophilic PTAO corona, which makes DOX more likely to diffuse from the cores.

### 2.2 In Vitro Cytotoxicity

**Figure 2:**
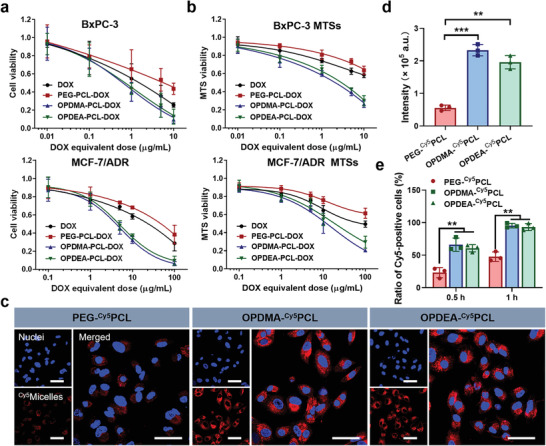
The in vitro cytotoxicity and cellular uptake of micelles. a) The in vitro cytotoxicity of DOX‐loaded micelles against adherent BxPC‐3 and MCF‐7/ADR cells determined by the MTT assay (48 h treatment). b) The in vitro cytotoxicity of DOX‐loaded micelles against multicellular tumor spheroids (MTSs) determined by the resazurin assay (48 h treatment). c) Confocal images of MCF‐7/ADR cells cultured with the PEG‐^Cy5^PCL, OPDMA‐^Cy5^PCL, and OPDEA‐^Cy5^PCL micelles at Cy5‐eq. dose of 0.5 µg mL^−1^ for 1 h. Cell nuclei stained with Hoechst 33 342 are shown in blue, and Cy5‐labeled micelles are shown in red. Scale bar: 25 µm. d) Quantification of Cy5 intensity in the confocal images using ImageJ. e) Time‐dependent cellular uptake of the PEG‐^Cy5^PCL, OPDMA‐^Cy5^PCL, and OPDEA‐^Cy5^PCL micelles in MCF‐7/ADR cells measured by flow cytometry. Cy5‐eq. dose: 0.5 µg mL^−1^.

The in vitro cytotoxicity of the micelle carriers, DOX‐loaded micelles, and free DOX was first evaluated on adherent cell culture models using the MTT (3‐[4,5‐dimethylthiazol‐2‐yl]‐2,5 diphenyl tetrazolium bromide) assay. The OPDMA‐PCL and OPDEA‐PCL carriers had minimal effects on cell proliferation in vitro, even at a concentration as high as 100 µg mL^−1^ (Figure S10, Supporting Information). After DOX loading, OPDMA‐PCL/DOX and OPDEA‐PCL/DOX showed higher cytotoxicity than free DOX and PEG‐PCL/DOX against a panel of cancer cell lines including 4T1, HepG2, HeLa, A549, BxPC‐3, and even drug‐resistant MCF‐7/ADR (**Figure** **2**a; Figure S11, Supporting Information). The IC50 values are listed in Table S2 (Supporting Information).

We also used the resazurin assay to further assess the cell‐killing effects of OPDMA‐PCL/DOX and OPDEA‐PCL/DOX on 3D multicellular tumor spheroids (MTSs). MTS models closely resemble solid tumors in many aspects, including the heterogeneous architecture, internal gradients of signaling factors, nutrients, and oxygenation. The cells in MTSs mimic intercellular and cell‐matrix interactions and exhibit greater chemotherapeutic resistance than monolayer‐cultured cells.^[^ ^21^ ^]^ On a dense MTS model of BxPC‐3 cell line (Figure 2b), the otherwise effective free DOX lost its cytotoxicity precipitously with an over 10‐fold increase in IC50, compared to that on the 2D model. Notably, OPDMA‐PCL/DOX and OPDEA‐PCL/DOX exhibited superior cytotoxicity, and their IC50 values were less than one‐fifth of those for DOX and PEG‐PCL/DOX. Moreover, free DOX showed moderate cytotoxicity against MCF‐7/ADR MTSs, whereas OPDMA‐PCL/DOX and OPDEA‐PCL/DOX still displayed potent cell‐killing effects. The corresponding IC50 values were 9.93 µg mL^−1^ and 16.48 µg mL^−1^, respectively, much lower than that of free DOX (41.68 µg mL^−1^) and PEG‐PCL/DOX (117.20 µg mL^−1^).

### 2.3 Cellular Uptake and Subcellular Distribution of PTAO‐PCL Micelles

Zwitterionic polymers are generally ultralow fouling and can shed potential protein or cell adsorption, thus suffering from unfavorable cellular uptake.^[^ ^22^ ^]^ Interestingly, OPDEA, albeit zwitterionic, can be easily internalized by cells.^[^ ^12^, ^14^ ^]^ We investigated whether the micellar formulations of OPDMA and OPDEA still retain this property. Fluorescently labeled micelles, OPDMA‐^Cy5^PCL, OPDEA‐^Cy5^PCL, and PEG‐^Cy5^PCL, were used to avoid interference from the release and fluorescence self‐quenching issues of DOX. Confocal microscopy showed bright Cy5 fluorescence inside PTAO micelle‐treated cells and significantly weaker Cy5 intensity in the PEG‐PCL group after 1 h incubation (Figure 2c,d). Flow cytometry analysis demonstrated that 66.13% and 60.57% of the cells treated with the OPDMA‐^Cy5^PCL and OPDEA‐^Cy5^PCL micelles were already Cy5 positive after 30‐min incubation, whereas only 23.13% were positive in the PEG‐^Cy5^PCL group. After 1 h incubation, the corresponding values increased to 95.40%, 93.17%, and 47.70%, respectively (Figure 2e; Figure S12, Supporting Information). These results indicated that PTAO‐PCL micelles could be internalized by cancer cells much faster than similarly antifouling PEG‐PCL micelles.

We then sought to clarify how the PTAO‐PCL micelles were internalized. We incubated MCF‐7/ADR cells at 4 °C or with a variety of endocytic inhibitors, including filipin III (an inhibitor of caveolae‐mediated endocytosis), chlorpromazine (an inhibitor of clathrin‐mediated endocytosis), wortmannin (an inhibitor of macropinocytosis), and cytochalasin D (an inhibitor of macropinocytosis/phagocytosis),^[^ ^23^ ^]^ and used flow cytometry to analyze their effects on cellular uptake. The results demonstrated that the cellular uptake of all the micelles was greatly blocked by low temperature, pointing to energy‐dependent pathways. While filipin III and chlorpromazine had no significant impacts on the internalization of OPDMA‐^Cy5^PCL and OPDEA‐^Cy5^PCL micelles, wortmannin and cytochalasin D exhibited similarly significant inhibitory effects (Figure S13, Supporting Information). These results suggested macropinocytosis‐mediated pathways for the cellular uptake of PTAO‐PCL micelles. In contrast, PEG‐^Cy5^PCL micelles were mainly internalized into cells via the clathrin‐ and caveolae‐related pathways.

**Figure 3:**
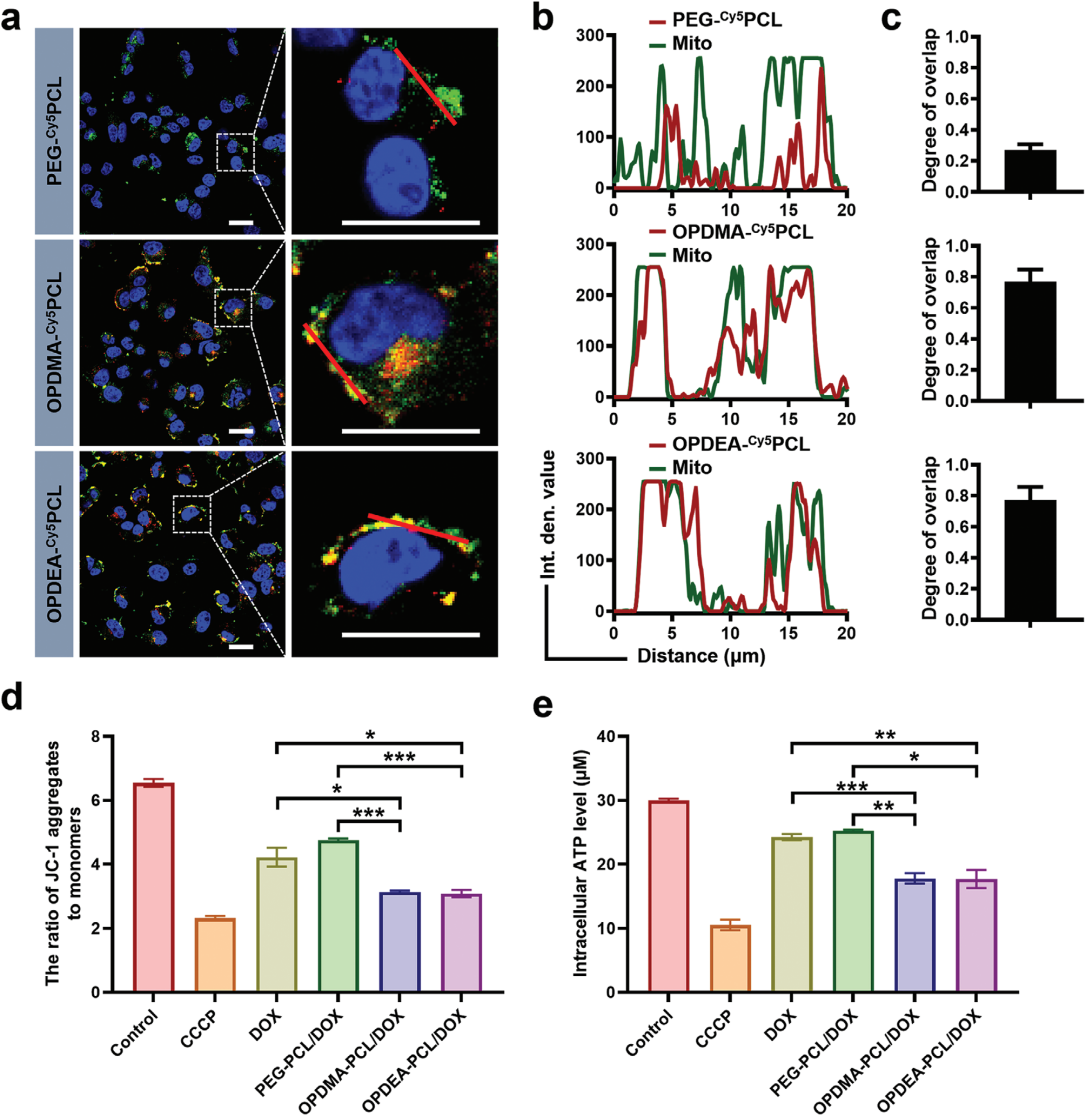
Intracellular trafficking of Cy5‐labeled micelles in MCF‐7/ADR cells. a) Colocalization of PEG‐^Cy5^PCL, OPDMA‐^Cy5^PCL, and OPDEA‐^Cy5^PCL with mitochondria after 2‐h incubation. Scale bar: 25 µm. b) Overlapping profiles of Cy5 fluorescence with MitoTracker Green fluorescence along the selected line across the cell (indicated by a red line in the zoomed‐in image). c) Manders’ correlation coefficients of Cy5 and MitoTracker Green calculated by pixel intensity using ImageJ. Cells were cultured with PEG‐^Cy5^PCL, OPDMA‐^Cy5^PCL, and OPDEA‐^Cy5^PCL (Cy5‐eq. dose: 0.1 µg mL^−1^) for 2 h. The Cy5 fluorescence was shown in red, and MitoTracker Green was in green. Scale bar: 25 µm. d) The JC‐1 assay of the ΔΨ_m_ in MCF‐7/ADR cells. The cells were incubated with DOX formulations at a DOX‐eq. dose of 0.5 µg mL^−1^ or CCCP (1 mM) for 2 h. e) Intracellular ATP levels of MCF‐7/ADR cells exposed to DOX formulations at a DOX‐eq. dose of 0.5 µg mL^−1^ or CCCP (1 × 10^−3^ m) for 12 h.

Subsequently, we investigated the subcellular distribution of the micelles using confocal microscopy. The PEG‐^Cy5^PCL micelles were found primarily localized to lysosomes (Figure S14, Supporting Information). In contrast, the Cy5 signals of the OPDMA‐^Cy5^PCL and OPDEA‐^Cy5^PCL micelles had weak correlations with the LysoTracker Green signals but overlapped the MitoTracker Green signals remarkably with the overlapping degrees as high as 76% and 77%, respectively (**Figure** **3**a–c). The DOX‐loaded Cy5‐free micelles also exhibited the same subcellular distribution profiles (Figure S15, Supporting Information). These results indicated that the intracellular PTAO‐PCL micelles were highly localized to mitochondria. The distinct subcellular localizations of the PTAO‐PCL and PEG‐PCL micelles are likely to result from the different endocytic pathways they underwent. While it is well appreciated that the clathrin‐mediated pathway may end up at lysosomes, how macropinosomes bypass the endosomal/lysosomal fusion but traffic to mitochondria, supposing the PTAO‐PCL micelles were still contained, is unknown, which we are working on.

As DOX may act on mitochondrial DNA to induce mitochondria disruption and cell apoptosis, mitochondria‐targeted DOX transportation may promise higher potency.^[^ ^24^ ^]^ We used JC‐1 assay to investigate the effects of PTAO‐PCL/DOX micelles on mitochondrial activity. The high membrane potential (ΔΨ_m_) of active mitochondria drives mitochondrial accumulation of the cationic lipophilic JC‐1 as J‐aggregates with red fluorescence (≈590 nm), whereas mitochondria depolarization‐induced cytosol distribution of monomeric JC‐1 leads to green fluorescence emission (529 nm).^[^ ^25^ ^]^ In order to avoid the interference of the DOX fluorescence (Ex: 488 nm, Em: 590 nm), the J‐aggregates were visualized by confocal microscopy, and the monomeric JC‐1 was detected using flow cytometry. As shown in Figure S16 (Supporting Information), bright red fluorescence of the J‐aggregates was observed in naïve cells. Upon treatment of PTAO‐PCL/DOX micelles, the red fluorescence from J‐aggregates declined significantly, weaker than that in the PEG‐PCL/DOX‐treated cells. The flow cytometry analysis showed that OPDMA‐PCL/DOX and OPDEA‐PCL/DOX micelles induced ≈76% of monomeric J‐1, significantly higher than that of DOX‐ (63%) and PEG‐PCL/DOX (36%)‐treated groups (Figure S17, Supporting Information). Moreover, the ratio of JC‐1 aggregates to monomers significantly decreased after MCF‐7/ADR cells were treated with PTAO‐PCL/DOX micelles, 73% and 65% of that of the DOX‐ or PEG‐PCL/DOX‐treated cells (Figure 3d). Accordingly, PTAO‐PCL/DOX micelles inhibited the ATP production; MCF‐7/ADR cells treated with PTAO‐PCL/DOX micelles had significantly lower intracellular ATP levels than those receiving DOX or PEG‐PCL/DOX (Figure 3e). Carbonyl cyanide 3‐chlorophenylhydrazone (CCCP) was here used as the positive control. These results demonstrate that PTAO‐PCL/DOX micelles may cause significant mitochondrial dysfunction.

### 2.4 Transcellular Transport of PTAO‐PCL Micelles

**Figure 4:**
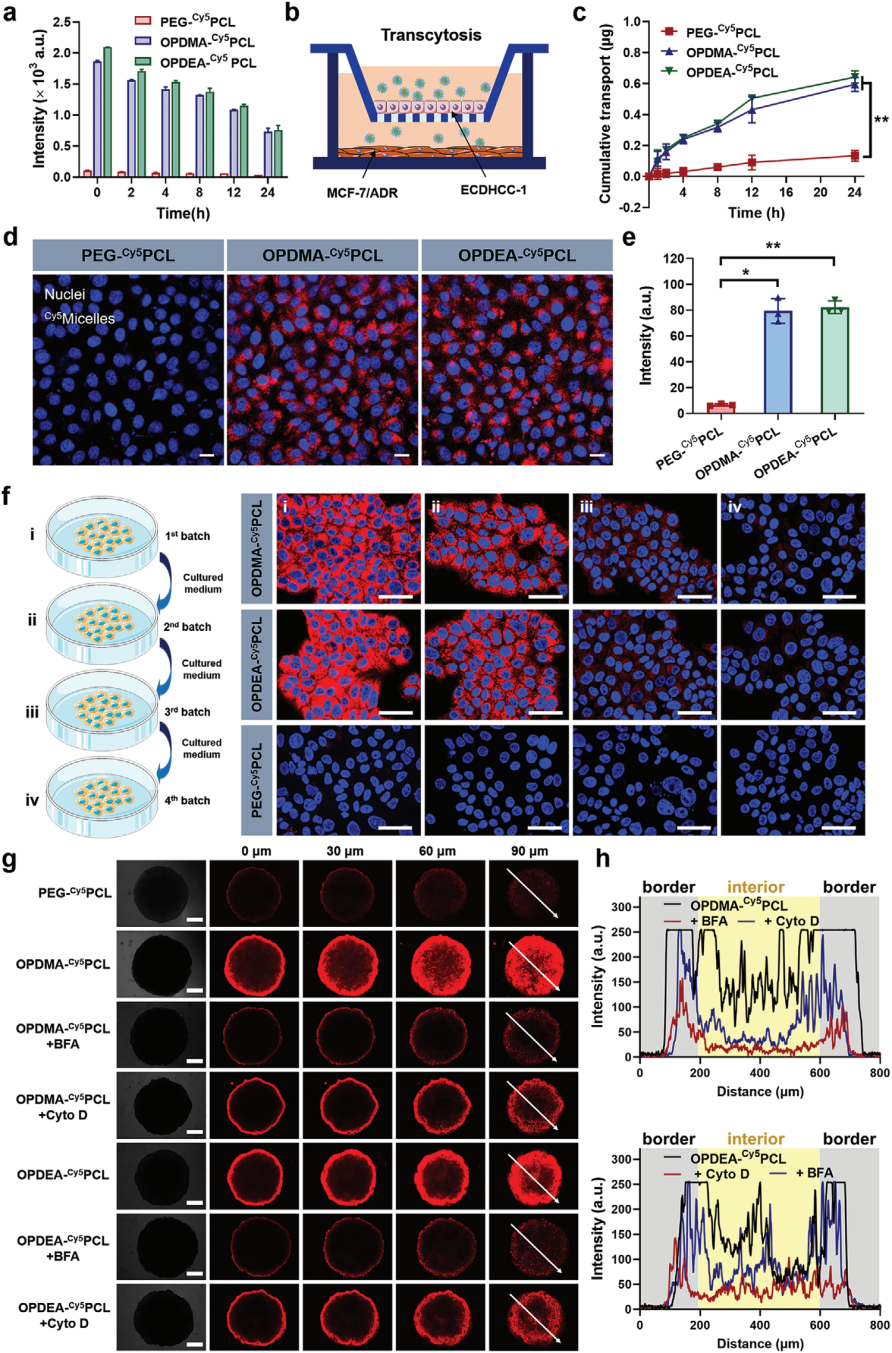
In vitro transcellular transport of micelles. a) The exocytosis kinetics measured by flow cytometry. ECDHCC‐1 cells were incubated with Cy5‐labeled micelles for 3 h and then re‐cultured in a fresh medium for timed intervals before flow cytometric analysis. b) Transepithelial transport of Cy5‐labeled micelles across the ECDHCC‐1 cell monolayer on a transwell membrane. c) Cumulative transport of the micelles. The Cy5‐labeled micelles at a Cy5‐eq. dose of 0.5 µg mL^−1^ were added onto the apical side, and the micelle concentrations in the basolateral compartment were measured at timed intervals by detecting the Cy5 fluorescence. d) Cellular uptake of the transported micelles in MCF‐7/ADR cells on the basolateral side after 24 h incubation. Cy5‐eq. dose: 0.5 µg mL^−1^. Scale bars: 100 µm. Cell nuclei were shown in blue and Cy5 in red. e) Cy5 fluorescence intensity of MCF‐7/ADR cells quantified by flow cytometry. f) Intercellular transport of Cy5‐labeled micelles between MCF‐7/ADR cells. The cells (first batch) were cultured with micelles at a Cy5‐eq. dose of 1 µg mL^−1^ for 6 h, washed with PBS, and imaged (i); the cells were then cultured in fresh medium for 12 h, and the medium was harvested to incubate the second batch of cells for 12 h, followed by washing and imaging (ii); the same procedures were implemented for another two rounds (iii and iv). Scale bar: 50 µm. g) Distribution of PEG‐^Cy5^PCL, OPDMA‐^Cy5^PCL, and OPDEA‐^Cy5^PCL in MCF‐7/ADR MTSs and the effects of endo/exocytosis inhibitors on the distribution. The MTSs were pretreated with or without cytochalasin D (5 × 10^−6^ m) or brefeldin A (90 × 10^−6^ m) for 10 h and then exposed to different DOX formulations for 4 h (Cy5 eq. dose of 5 µg mL^−1^). The MTSs were washed twice with 10% heparin‐containing PBS and visualized using confocal microscopy in Z‐stacks with 30 µm intervals. The midplane is denoted as 0 µm. Scale bar: 250 µm. h) The Cy5 fluorescence intensity along with the randomly selected white arrows across OPDMA‐^Cy5^PCL and OPDEA‐^Cy5^PCL‐treated MTSs. ^*^ *p* < 0.05, ^**^ *p* < 0.01.

To study the transcellular transport of PTAO‐PCL micelles, we performed five sets of experiments involving different cellular environments. As transcellular transport involves exocytosis as well as endocytosis, in the first test, we attempted to investigate the exocytosis kinetics of the internalized PTAO‐PCL micelles in hepatocellular carcinoma‐derived endothelial (ECDHCC‐1) cells. The flow cytometry measurement indicated that ECDHCC‐1 cells gradually secreted PTAO‐PCL micelles, with 24 h secretion ratios reaching up to 61% and 64% for OPDMA‐^Cy5^PCL and OPDEA‐^Cy5^PCL, respectively (**Figure** **4**a). These results imply that along with a large portion of the PTAO‐PCL micelles exocytosed, a small portion would retain in the cells to allow drug actions.

Then, we sought to measure the transepithelial transport rate of the micelles crossing the monolayer of vascular endothelial cells on a transwell membrane (Figure 4b). ECDHCC‐1 cells were cultured on the membrane of the apical side of a transwell device to generate a cell monolayer with a transepithelial electrical resistance over 500 Ω cm^2^.^[^ ^26^ ^]^ PTAO‐^Cy5^PCL and PEG‐^Cy5^PCL micelles were then applied to the apical side, and the micelle concentrations in the basolateral compartment were measured to evaluate the transcellular transport capacity. As shown in Figure 4c, PEG‐^Cy5^PCL micelles hardly crossed the monolayer, while the PTAO‐^Cy5^PCL micelles traveled rapidly to the basolateral side. The transcellular transport rates of OPDMA‐^Cy5^PCL and OPDEA‐^Cy5^PCL were 75 and 81 ng h^−1^ cm^−2^, respectively, significantly higher than that of PEG‐^Cy5^PCL (17 ng h^−1^ cm^−2^). This transcellular transport of PTAO–PCL micelles was remarkably suppressed at a low temperature, suggesting an energy‐dependent process. Since the macropinocytosis‐mediated pathway was involved in the endocytosis of the PTAO‐PCL micelles, inhibiting micropinocytosis by using wortmannin or cytochalasin D could drastically retard the transepithelial transport. Moreover, disrupting Golgi apparatus by monensin^[^ ^27^ ^]^ or blocking Golgi protein transport by brefeldin A^[^ ^28^ ^]^ both decreased the transport rates of PTAO‐PCL micelles, pointing to a Golgi apparatus‐involved transcytosis process (Figure S18, Supporting Information).

In the next experiment, we aimed to investigate if the micelles undergoing the transcellular transport could still be internalized by cancer cells. We set up the experiment conditions as the same as the previous one but seeded MCF‐7/ADR cells on the basolateral side of the device. Confocal microscopy images demonstrated extensive internalization of the PTAO‐^Cy5^PCL micelles by MCF‐7/ADR cells, as opposed to the barely observable fluorescence in the PEG‐^Cy5^PCL micelle‐treated cells (Figure 4d). An 11.9‐ and 12.3‐fold higher fluorescence intensity in the OPDMA‐^Cy5^PCL and OPDEA‐^Cy5^PCL groups than that in the PEG‐^Cy5^PCL group was determined by flow cytometry analysis (Figure 4e). These results suggest that PTAO‐PCL micelles may undergo transcytosis‐mediated serial transport.

To validate this transport mode of PTAO‐PCL micelles, we then performed an “infection” assay.^[^ ^29^ ^]^ MCF‐7/ADR cells were treated with micelles for 6 h, followed by another 12‐h incubation in the micelle‐free medium. Afterward, the medium was collected for incubation with another batch of cells. These procedures were performed for three rounds in total. As shown in Figure 4f, Cy5 signals could be observed in all the batches of cells in the PTAO‐^Cy5^PCL groups in a batch‐dependent fashion. In contrast, only the first batch of cells in the PEG‐^Cy5^PCL group fluoresced weakly in the Cy5 channel. The induction of transcytosis by PTAO‐PCL micelles is likely to arise from the rapid cellular internalization via macropinocytosis, followed by the translocation from the leak macropinosomes to the Golgi apparatus for efficient exocytosis.^[^ ^30^ ^]^

As a final test, we challenged the PTAO‐PCL micelles with MTS models, where the abundant matrix may hinder the delivery of nanomedicines. MCF‐7/ADR MTSs with a diameter of ≈1 mm were cultured with OPDMA‐^Cy5^PCL, OPDEA‐^Cy5^PCL, or PEG‐^Cy5^PCL micelles for 4 h and then transferred onto glass‐bottomed Petri dishes for imaging (Figure 4g). Weak Cy5 fluorescence of the PEG‐^Cy5^PCL micelles could be observed only around the periphery of the MTSs and hardly in the interior. In contrast, the whole MTSs receiving the OPDMA‐^Cy5^PCL or OPDEA‐^Cy5^PCL micelles fluoresced brightly, with the fluorescence intensity decreasing gradually from the periphery to the center. A linescan analysis of fluorescence intensity as a function of distance from the periphery evidenced that the MTSs treated with OPDMA‐^Cy5^PCL or OPDEA‐^Cy5^PCL micelles had a much higher fluorescence intensity inside the tumor spheroids than those treated with PEG‐^Cy5^PCL micelles (Figure 4h). Pretreatment of MTSs with wortmannin or brefeldin A abolished the PTAO micelles’ penetrating capability and confined them to the surface of the spheroids (Figure 4g), further strengthening the macropinocytosis‐involved transcytotic pathways. The penetration phenomena in MTSs were further confirmed using the DOX‐loaded micelles (Figure S19, Supporting Information). These results well explained the potent cytotoxicity of the DOX‐loaded PTAO‐PCL micelles against MTSs. Accordingly, adding these endo‐ and exocytosis inhibitors significantly decreased the cytotoxicity of the DOX‐loaded PTAO‐PCL micelles against MTSs (Figure S20, Supporting Information).

### 2.5 Blood Clearance, Biodistribution, and In Vivo Penetration

RBC hitchhiking has been demonstrated as an efficient approach for drug delivery that can prolong blood circulation and enhance drug accumulation in the targets.^[^ ^31^ ^]^ Despite the polyzwitterionic nature, OPDMA and OPDEA on the micelles’ surface could adhere to RBCs. Compared with free DOX or PEG‐PCL/DOX micelles, RBCs incubated with OPDMA‐PCL/DOX or OPDEA‐PCL/DOX micelles showed stronger DOX fluorescence, which overlapped well with the ^FITC^WGA‐labeled RBC membrane (Figure S21, Supporting Information). Additionally, adsorption of PTAO‐PCL/DOX micelles did not significantly change RBCs' morphology, integrity, and dispensability. Therefore, PTAO‐PCL micelles may hitchhike on RBCs via cell binding to avoid the clearance by the mononuclear phagocyte system (MPS), which promises favorable pharmacokinetics and biodistribution profiles.

**Figure 5:**
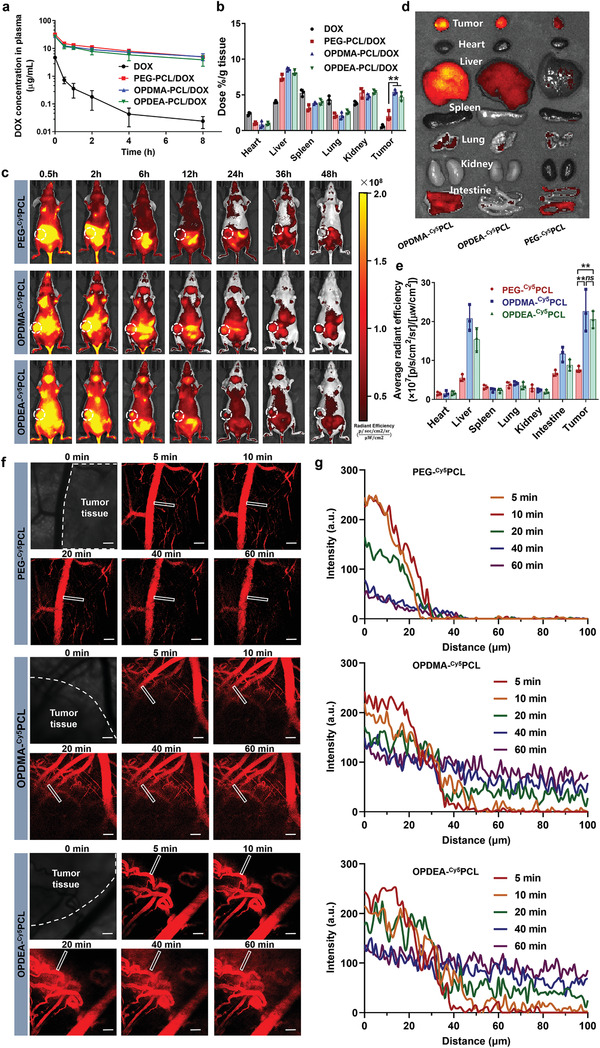
Blood clearance, biodistribution, and tumor penetration of micelles in MCF‐7/ADR tumor‐bearing mice. a) The blood clearance kinetics of DOX after intravenous administration of DOX, PEG‐PCL/DOX, OPDMA‐PCL/DOX, or OPDEA‐PCL/DOX at a DOX‐eq. dose of 4 mg kg^−1^ (*n* = 3). b) Distribution of DOX in tumors and major organs at 24 h post‐injection of DOX, PEG‐PCL/DOX, OPDMA‐PCL/DOX, or OPDEA‐PCL/DOX. c) In vivo real‐time imaging of tumor‐bearing mice after a single intravenous injection of PEG‐^Cy5^PCL, OPDMA‐^Cy5^PCL, or OPDEA‐^Cy5^PCL (Cy5‐eq. dose of 0.5 mg kg^−1^). The tumor regions were circled in white. d) Ex vivo imaging of tumors and organs (heart, liver, spleen, lung, and kidney) excised from the above mice. e) Quantification of average Cy5 fluorescence intensity in tumors and organs. f) Confocal imaging of time‐resolved extravasation and penetration of PEG‐^Cy5^PCL, OPDMA‐^Cy5^PCL, and OPDEA‐^Cy5^PCL into MCF‐7/ADR tumors (Cy5‐eq. dose of 0.5 mg kg^−1^). BALB/c nude mice were inoculated with MCF‐7/ADR cells (5 × 10^7^ cells per mouse) near the subcutaneous venous vessel in the abdomen. When the tumor volume reached ≈40 mm^3^, the mice were anesthetized, and the tumor was exposed and fixed for imaging. Scale bar: 50 µm. g) Cy5 fluorescence intensity of PEG‐^Cy5^PCL, OPDMA‐^Cy5^PCL, and OPDEA‐^Cy5^PCL as a function of the distance from a blood vessel into an in‐depth tumor region marked by the rectangular frames. NS, no significance; ^**^ *p* < 0.01.

We first studied the blood clearance kinetics of PTAO‐PCL/DOX micelles. DOX or DOX‐loaded micelles were intravenously injected into mice, and blood samples were drawn at timed intervals to quantify the DOX concentrations using HPLC (**Figure** **5**a). After administration, free DOX was cleared rapidly from the bloodstream, with only 0.5% of the given dose remaining after 8 h, whereas the micelle formulations circulated long in blood, with over 14% remaining after 8 h. Moreover, the OPDMA‐PCL/DOX and OPDEA‐PCL/DOX micelles had similar blood clearance kinetics to that of the long‐circulating PEG‐PCL/DOX micelles, with comparable AUC values and half‐life times (Table S3, Supporting Information). More interestingly, despite the similar pharmacokinetics, ex vivo biodistribution analysis at 24 h post‐injection shows that OPDMA‐PCL/DOX and OPDEA‐PCL/DOX micelles exhibited 2.7‐ and 2.3‐fold higher DOX accumulation in tumors than PEG‐PCL/DOX, respectively (Figure 5b). The superior tumor accumulation of PTAO‐PCL/DOX over PEG‐PCL/DOX may be attributed to the ability of PTAO‐PCL micelles to induce transcellular transport, which allows PTAO‐PCL/DOX to bypass the enhanced permeability and retention effect^[^ ^32^ ^]^ and enter tumor tissues via transcytosis‐mediated extravasation.

We further studied the in vivo real‐time biodistribution of PTAO‐PCL micelles in BALB/c nude mice bearing MCF‐7/ADR xenografts. Mice receiving a single injection of Cy5‐labeled micelles (Cy5‐eq. dose of 0.2 mg kg^−1^) were imaged using a near‐infrared fluorescence imaging system (Figure 5c). Significant fluorescence contrast between tumors and tumor‐adjacent tissues was detected in the OPDMA‐^Cy5^PCL and OPDEA‐^Cy5^PCL groups essentially throughout the whole experimental period but did not occur in the PEG‐^Cy5^PCL group. After 48 h post‐treatment, the mice were sacrificed, and the major organs and tumor tissues were excised for ex vivo imaging (Figure 5d). Tumors from the OPDMA‐^Cy5^PCL and OPDEA‐^Cy5^PCL groups fluoresced brighter than any other organs or tissues, as well as those from the PEG‐^Cy5^PCL group (Figure 5e). These results demonstrated that the PTAO‐PCL micelles could accumulate efficiently in tumors.

**Figure 6:**
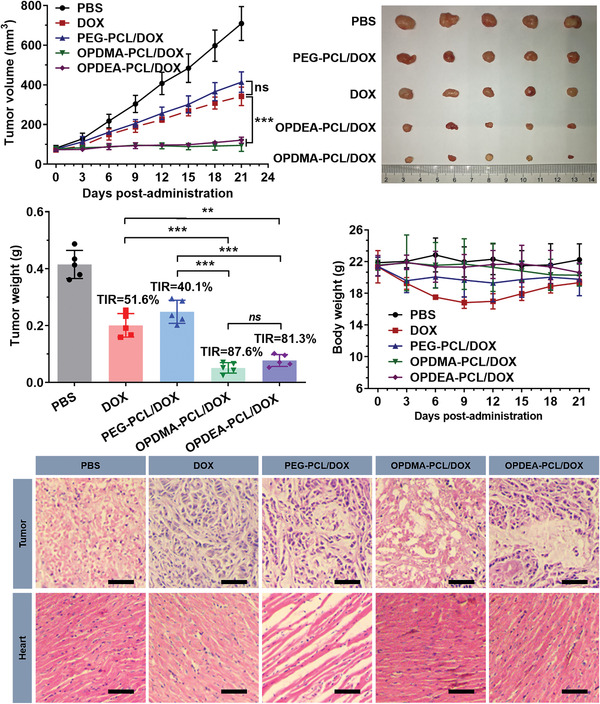
Antitumor activities of DOX‐loaded PTAO‐PCL micelles against orthotopic MCF‐7/ADR tumors. a) Tumor growth curves of the mice. b) Photographs of the tumors resected at the end of the experiment. c) Averaged tumor weight of each group at the experimental endpoint and tumor inhibition rates (TIR). d) Body weight variation of the mice during the experiment. e) Representative histological features of the tumors and hearts. The 10‐µm‐thick tissue sections were stained with hematoxylin‐eosin and observed using light microscopy. Scale bar: 50 µm. ^*^ *p* < 0.05, ^**^ *p* < 0.01, ^***^ *P* < 0.001.

Next, we validated the in vivo tumor penetration ability of PTAO‐PCL micelles. Mice bearing MCF‐7/ADR tumors of ≈40 mm^3^ were intravenously injected with OPDMA‐^Cy5^PCL, OPDEA‐^Cy5^PCL, or PEG‐^Cy5^PCL micelles, and the tumor regions were imaged using confocal microscopy. As shown in **Figure** **6**f, OPDMA‐^Cy5^PCL and OPDEA‐^Cy5^PCL gradually extravasated from the blood vessels and infiltrated into the distal tumor sites. In contrast, PEG‐^Cy5^PCL micelles were essentially confined in the blood vessels even after 60 min post‐injection. The line‐scan analysis showed that OPDMA‐^Cy5^PCL and OPDEA‐^Cy5^PCL could diffuse into tumor tissues as far as 100 µm away from the vessels while the PEG‐^Cy5^PCL signal was already undetectable at distances 40 µm away from the vessels (Figure 6g). As a result, the overall fluorescence intensity of PTAO‐PCL micelles along the selected line increased linearly with time, reaching over 27 times the intensity of PEG‐^Cy5^PCL micelles at 60 min (Figure S22, Supporting Information). These results demonstrated that the PTAO‐PCL micelles could extravasate quickly into tumor tissues and possess superior tumor permeability over PEG‐PCL micelles.

### 2.6 In Vivo Antitumor Activity

The antitumor activities of the PTAO‐PCL/DOX micelles were evaluated in an MCF‐7/ADR orthotopic tumor model. Tumor‐bearing mice were intravenously administered with PBS, DOX, PEG‐PCL/DOX, OPDMA‐PCL/DOX, or OPDEA‐PCL/DOX at DOX equivalent dose of 4 mg kg^−1^ every 3 days for a total of five treatments (Figure 6). Compared with the PBS group, each DOX formulation led to obvious tumor growth inhibition. Nonetheless, the antitumor efficacy of DOX and PEG‐PCL/DOX was moderate, with tumor inhibition rates (TIRs) of 51.6% and 40.1%, respectively. In contrast, OPDMA‐PCL/DOX and OPDEA‐PCL/DOX basically suppressed tumor growth during the whole experimental period with TIRs as high as 87.6% and 81.3%, respectively (Figure 6a–c; Figure S23, Supporting Information). Histological examination of the tumor tissue sections showed abundant apoptotic cells with features including extensive vacuolization, severe nucleus shrinkage, and decreased cellularity in the OPDMA‐PCL/DOX and OPDEA‐PCL/DOX groups. In comparison, much fewer apoptotic events were observed in the DOX and PEG‐PCL/DOX groups (Figure 6e). These results demonstrated that compared with the traditional PEGylated micelles, PTAO micelles, with the efficient tumor‐penetration ability and remarkable mitochondrial targeting ability, could reverse cancer cells’ MDR and thus achieve high therapeutic outcomes.

Importantly, no significant change in body weight occurred on the mice receiving OPDMA‐PCL/DOX and OPDEA‐PCL/DOX treatments, whereas the mice treated with DOX suffered from severe weight loss (up to 20%), though gradually rebounded after intraperitoneal administration with 1% (w/v) glucose solution (Figure 6d). Histological analysis showed no noticeable damages or lesions in the major organs resected from the mice in the PEG‐PCL/DOX, OPDMA‐PCL/DOX, and OPDEA‐PCL/DOX groups, whereas free DOX caused gross damage to cardiomyocytes, evidenced by the disordered and even fractured cardiac muscle fibers (Figure 6e; Figure S24, Supporting Information). These results indicated the high biosafety of the DOX‐loaded PTAO‐PCL micelles.

## 3 Conclusion

In summary, we have presented PTAO‐PCL micelles for efficient drug delivery into tumors. Due to the zwitterionic nature and unique cell membrane affinity, the PTAO micelles exhibited long blood circulation, efficient tumor accumulation and penetration, and fast cellular internalization. Moreover, the DOX‐loaded PTAO micelles could target the mitochondria and lead to mitochondrial dysfunction, thus reversing the multidrug resistance of tumor cells. These abilities enabled potent therapeutic efficacy against MCF‐7/ADR tumors. We envision that these PTAO polymers, as superior alternatives to PEG, may be applied to other drug delivery platforms such as liposomes and dendrimers for higher therapeutic outcomes.

## Conflict of Interest

The authors declare no conflict of interest.

## Supporting information

Supporting Information

## Data Availability

The data that support the findings of this study are available in the supplementary material of this article.
