# Supplementary material for: Multipotent Poly(Tertiary Amine‐Oxide) Micelles for Efficient Cancer Drug Delivery
Source: Adv Sci (Weinh). 2022 Feb 20;9(12):2200173. doi: 10.1002/advs.202200173 (PMC9036005; doi:10.1002/advs.202200173)
Supplement: Supplementary file 1 — Supporting Information [file ADVS-9-2200173-s001.pdf]

Multipotent Poly(tertiary amine-oxide) Micelles for Efficient Cancer Drug Delivery

Jiajia Xiang†, Yihuai Shen†, Yifan Zhang†, Xin Liu, Quan Zhou, Zhuxian Zhou, Jianbin Tang, Shiqun Shao, Youqing Shen**

J.J. Xiang, Y.F. Zhang, Z.X. Zhou, J.B. Tang, S.Q. Shao, Y.Q. Shen
Zhejiang Key Laboratory of Smart BioMaterials and Center for Bionanoengineering, College of Chemical and Biological Engineering, Zhejiang University, Hangzhou 310027, China.
ZJU-Hangzhou Global Scientific and Technological Innovation Center, Hangzhou 311215, China.
E-mail: shiqun.shao@zju.edu.cn (S.Q. Shao), shenyq@zju.edu.cn (Y.Q. Shen)
J.J. Xiang, Y.H. Shen, Y.F. Zhang, Z.X. Zhou, J.B. Tang, S.Q. Shao, Y.Q. Shen
Key Laboratory of Biomass Chemical Engineering of the Ministry of Education, College of Chemical and Biological Engineering, Hangzhou, Zhejiang University, Hangzhou 310027, China.
E-mail: shiqun.shao@zju.edu.cn, shenyq@zju.edu.cn
Xin Liu
Department of Orthopaedic Surgery, Sir Run Run Shaw Hospital, Medical College of Zhejiang University, Hangzhou 310016, China.
Q. Zhou
School of Basic Medical Sciences, Zhejiang University, Hangzhou 310058, China.
† These authors contributed equally to this work.

Materials and methods

Materials

5-Amino-1-pentanol, di-*tert*-butyl dicarbonate, δ-caprolactone (δ-CL), diphenyl phosphate (DPP), 2-bromoisobutyryl bromide, *N*,*N*-dimethylaminoethyl methacrylate (DMA), *N*,*N*-diethylaminoethyl methacrylate (DEA), 3-chloroperbenzoic acid (mCPBA), CuBr, *N*,*N*,*N*,*N*-pentamethyldiethylenetriamine (PMDETA), BOC-Glycine, 4-(dimethylamino)pyridine (DMAP) and trifluoroacetic acid (TFA) were purchased from Energy Chemical (Shanghai, China). Doxorubicin hydrochloride salt (DOX·HCl), propidium iodide (PI), and methoxypoly(ethylene glycol) with a

molecular weight of ~5000 (mPEG5k-OH) were purchased from Sigma-Aldrich (Shanghai, China). Cy5-NHS was purchased from Xi'an Ruixi Biological Technology Co., Ltd. (Xi'an, China). Other chemical reagents and solvents were purchased from Sinopharm Chemical Reagent Co. Ltd. (Shanghai, China). Dulbecco's Modified Eagle Medium (DMEM) and Roswell Park Memorial Institute-1640 (RPMI-1640) cell culture medium, fetal bovine serum (FBS), and 0.25% trypsin-EDTA were purchased from Gibco (USA). 3-(4,5-Dimethylthiazol-2-yl)-2,5-diphenyltetrazolium bromide (MTT) was purchased from Dalian Meilun Biotech. Inc. (Dalian, China).

Synthesis of 5-(Boc-amino)-1-pentanol

To a solution of 5-amino-1-pentanol (8.25 g, 80 mmol) in 150 mL anhydrous CH_2Cl_2, di-*tert*-butyl dicarbonate (20.95 g, 96 mmol) in 50 mL CH_2Cl_2 was added dropwise at 0 ºC with vigorous stirring. Afterward, the mixture was warmed to room temperature and stirred for another 2 h. After removing the solvent by evaporation, 300 mL ethyl acetate was added to dissolve the residues. The organic solution was washed thrice with brine and then dried over anhydrous Na_2SO_4. After removing the solvent, 5-(Boc-amino)-1-pentanol was obtained as a colorless oil (12.4 g, yield: 76.2%). ^{1}H NMR (400 MHz, $CDCl_3$) δ 4.62 (s, 1H), 3.64 (t, J = 5.6 Hz, 2H), 3.26 – 2.98 (m, 2H), 1.65 – 1.33 (m, 15H).

Synthesis of Boc-amino terminated poly (ε-caprolactone) (Boc-NH-PCL-OH)

Freshly distilled δ-CL (13.13 g, 115 mmol) and 5-(Boc-amino)-1-pentanol (0.51 g, 2.5 mmol) were dissolved in 20 mL anhydrous toluene under argon atmosphere. DPP (0.63 g, 2.5 mmol) in 3 mL toluene was injected to initiate the polymerization. After 4 h polymerization at 30°C, the reaction solution was poured into 200 mL cold ether. The precipitate was isolated by filtration, washed with ether, and dried under vacuum to give Boc-NH-PCL-OH (11.7 g, yield: 85.8%) as a white solid. $M_{n,\ NMR}$, 5500 g mol^{-1}; Mw/Mn, 1.06. ^{1}H NMR (400 MHz, $CDCl_3$) δ 4.06 (t, J = 6.7 Hz, 94H), 3.65 (t, J = 6.5 Hz, 2H), 2.31 (t, J = 7.5 Hz, 92H), 1.70 – 1.57 (m, 188H), 1.44 (s, 9H), 1.43 – 1.33 (m, 94H).

Synthesis of ATRP macroinitiator PCL-Br

Boc-NH-PCL-OH (0.50 g, 0.45 mmol) was mixed with triethylamine (0.34 g, 3.38 mmol) in 50 mL anhydrous CH_2Cl_2 in an ice bath. 2-Bromoisobutyryl bromide (0.52 g, 2.25 mmol) in 20 mL CH_2Cl_2 was then added dropwise under Argon atmosphere. After stirring at 0°C for 2 h, the reaction was allowed to warm to room temperature and stirred overnight. The reaction mixture was filtered and concentrated by rotary evaporation, and the residue was poured into cold methanol. The precipitate was isolated, washed with cold methanol and ether, and dried under vacuum to give PCL-Br (2.21 g, yield: 86.1%) as a white solid. ^{1}H NMR (400 MHz, $CDCl_3$) δ 4.06 (t, J = 6.7 Hz, 94H), 3.69 (t, J = 6.5 Hz, 2H), 2.30 (t, J = 7.5 Hz, 92H), 1.92 (s, 6H), 1.68 – 1.59 (m, 188H), 1.41 (s, 9H), 1.40 – 1.32 (m, 94H).

Synthesis of *N*-oxide-*N,N*-dimethylaminoethyl methacrylate (ODMA) and *N,N*-diethylaminoethyl methacrylate (ODEA)

N,N-dimethylaminoethyl methacrylate (DMA, 3.14 g, 20 mmol) was dissolved in 80 mL anhydrous CH_2Cl_2 in an ice bath. mCPBA (5.18 g, 30 mmol) was added portion-wise within 30 min with vigorous stirring. Afterward, the solution was allowed to warm to room temperature and stirred for another 2 h. After removing the solvent by evaporation, the residues were purified using a neutral alumina column, affording ODMA (3.1 g, yield: 89.5%) as an off-white waxy solid. The pure ODMA was stored at -20 °C. ^{1}H NMR (400 MHz, $CDCl_3$) δ 6.11 (s, 1H), 5.62 (s, 1H), 4.78 (t, 2H), 3.60 – 3.56 (t, 2H), 3.28 (s, 6H), 1.94 (s, 3H).

Similarly, N,N-diethylaminoethyl methacrylate (ODEA) was obtained as an off-white waxy solid (yield: 86.4%). ^{1}H NMR (400 MHz, $CDCl_3$) δ 6.08 (s, 1H), 5.60 (s, 1H), 4.74-4.70 (t, 2H), 3.46 – 3.43 (t, 2H), 3.31 – 3.24 (m, 4H), 1.92 (s, 3H), 1.30 (t, J = 7.2 Hz, 6H).

Synthesis of OPDMA-PCL and OPDEA-PCL

OPDMA-PCL was synthesized *via* atom transfer radical polymerization (ATRP). PCL-Br (0.50 g, 0.20 mmol) and ODMA (0.50 g, 2.9 mmol) were added to a mixture of CuBr (28.69 mg, 0.20 mmol) and PMDETA (34.66 mg, 0.20 mmol) in 6 mL of DMF. The mixture was degassed by three freeze–pump–thaw cycles, and then placed in a preheated oil bath at 40 °C. After 24 h, the reaction was quenched by exposing to

air and dialyzed against DMSO (500 mL×3) and DI water (500 mL×3). The solution was lyophilized to give OPDMA-PCL (0.67 g, yield: 67.0%) as a pale-yellow solid. ^{1}H NMR (400 MHz, $CDCl_3$) δ 4.45 (s, 60H), 3.98 (t, *J* = 6.4 Hz, 94H), 3.49 (s, 62H), 3.15 (s, 180H), 2.27 (t, *J* = 7.2 Hz, 92H), 2.08 – 1.39 (m, 254H), 1.36 (s, 9H), 1.30 (d, *J* = 7.0 Hz, 92H), 0.91 (d, *J* = 61.4 Hz, 92H).

OPDEA-PCL was obtained similarly as a pale-yellow solid (yield: 62.0%). ^{1}H NMR (400 MHz, $CDCl_3$) δ 4.41 (s, 48H), 3.98 (t, *J* = 6.4 Hz, 94H), 3.38 (s, 98H), 2.27 (t, *J* = 7.2 Hz, 92H), 1.54 (m, *J* = 15.0 Hz, 386H), 1.36 (s, 9H), 1.32 – 1.24 (m, 80H).

Synthesis of Cy5-labeled OPDMA-PCL (OPDMA-^{Cy5}PCL) and OPDEA-PCL (OPDEA-^{Cy5}PCL)

OPDMA-PCL (0.1 g) was dissolved in a mixture of CH_2Cl_2 (2 mL) and TFA (1 mL). After stirring at room temperature for 3 h, the solvent was removed by rotary evaporation. The residues were re-dissolved in 2 mL DMF, and then Cy5-NHS (1 mg) and Et_3N (0.2 mL) were added. The solution was stirred overnight at room temperature in the dark and then dialyzed against DMSO (500 mL×3) for 24 h and then against DI water (2 L×3) for 24 h. OPDMA-^{Cy5}PCL was further purified through a Sephadex column to remove any residual Cy5 molecules and then lyophilized to afford a blue powder (72.4 mg, yield: 72.4%).

OPDEA-^{Cy5}PCL was obtained similarly as a blue powder (yield: 68.5%).

Synthesis of PEG-PCL

δ-CL (0.50 g, 4.38 mmol) and mPEG$_{5k}$-OH (0.50 g, 0.10 mmol) was dissolved in 2 mL anhydrous toluene under argon atmosphere. DPP (25.19 mg, 0.10 mmol) in 1 mL of toluene was injected to initiate the polymerization. After 4 h polymerization at 30°C, the reaction solution was poured into 100 mL cold ether. The precipitate was isolated by filtration, washed with ether, and dried under vacuum to give PEG-PCL (0.54 g, yield: 54.0%) as a white solid. ^{1}H NMR (400 MHz, $CDCl_3$) δ 4.05 (t, *J* = 6.7 Hz, 96H), 3.64 (s, 536H), 3.37 (s, 3H), 2.30 (t, *J* = 7.5 Hz, 96H), 1.68 – 1.60 (m, 192H), 1.38 (m, *J* = 12.6 Hz, 96H).

Synthesis of Cy5-labeled PEG-PCL (PEG-^{Cy5}PCL)

PEG-PCL (0.50 g , 0.05 mmol), BOC-Glycine (0.09 g, 0.50 mmol) and DMAP (0.05 g, 0.41 mmol) were dissolved in 50 mL anhydrous CH_2Cl_2 at 0 °C followed by the addition of DCC (0.10 g, 0.50 mmol) in 5 mL CH_2Cl_2. The reaction mixture was then warmed to room temperature and stirred overnight. After filtration, the solvent was removed by evaporation, and the residues were poured into cold methanol. The precipitate was isolated and washed with cold methanol and ether. After drying under vacuum, PEG-PCL-NHBoc was obtained as a white solid (0.38g, yield: 76.0%).

PEG-^{Cy5}PCL was synthesized as described above (yield: 71.0%).

Fabrication and characterization of DOX-loaded micelles

Doxorubicin (DOX) hydrochloride salt (1 mg) was dissolved in 1 mL of DMSO followed by the addition of triethylamine (10 μL). After stirring in the dark for 4 h, OPDMA-PCL or OPDEA-PCL (5 mg) was added and stirred for another 1 h. The solution was then added dropwise into 5 mL DI water and stirred for 30 min. Then the mixture was dialyzed against PBS (pH 8.0, 1 L×3) and DI water (1 L×2). As the unloaded DOX formed large aggregates in the micelle solution, the DOX aggregates could be removed using membrane filters with a pore diameter of 220 nm while keeping the micelles due to their small sizes. The DOX drug loading content and loading efficiency were analyzed by measuring the UV absorbance at 480 nm in DMSO and calculated using the standard curve. The micelles' volume-averaged sizes and zeta potentials were measured by dynamic light scattering (DLS), and the morphology of the micelles was observed by TEM after being negatively stained with 1% uranium acetate.

Critical micelle concentration (CMC) determination

The CMC of OPDMA-PCL, OPDEA-PCL, and PEG-PCL was determined following the reported protocol. A defined amount of Nile Red in CH_2Cl_2 was added to a series of vials, and the solvent was evaporated. The amount was chosen to give the final solutions a Nile Red concentration of 8.0×10-7 M. OPDMA-PCL, OPDEA-PCL, and PEG-PCL of serial concentrations from 0.1 to 250 μg/mL were then added. The vials were shaken at 37°C overnight in the dark at a speed of 200 rpm.

The fluorescence intensity of each solution at 620 nm was measured upon excitation of 579 nm using a multiwell plate reader (SpectraMax M2E, Molecular Devices Inc., San Jose, CA, USA). The CMC values were defined as the intersection of the two fitting lines of the plots of fluorescence intensity versus the micelle concentration.

Stability test of micelles in different mediums

PEG-PCL/DOX, OPDMA-PCL/DOX, or OPDEA-PCL/DOX micelles were incubated with DI water, PBS (10 mM, pH 7.4), or DMEM medium containing 10% FBS at 37 ℃. At timed intervals, 200 μL of each micelle solution was withdrawn for DLS measurement. The variation of particle sizes in each group was recorded and plotted as a function of time.

***In vitro* DOX release from DOX-loaded micelles**

In vitro DOX release from PEG-PCL/DOX, OPDMA-PCL/DOX, and OPDEA-PCL/DOX micelles were analyzed by a dialysis method. 2 mL of micelle solutions with 200 μg/mL of DOX were applied to a dialysis bag (MWCO 3500). The dialysis bag was then immersed in 50 mL of PBS buffer (pH 7.4 or 5.0) at 37 °C and gently agitated at a speed of 150 rpm in the dark. 0.5 mL of the PBS medium was sampled at timed intervals and re-added an equal amount of fresh medium. The DOX concentration in the medium was determined by UV absorption at 480 nm, and the accumulative release amount was calculated.

Establishment of multicellular tumor spheroids (MTSs)

MTSs of MCF-7/ADR cells were established as previously reported.[1] MCF-7/ADR cells were suspended in DMEM medium containing 0.24% (w/v) methylcellulose and 10% (v/v) FBS at a density of 6 × 10^5 cells/mL. Cell suspensions (20 μL) were dropped on the lids of 96-well plates as uniform droplets, and then 100 μL of PBS was added to each well to keep the droplets moist. After incubation for 48 h, dense spheroids were formed with a diameter of ~0.3 mm. The spheroids were then transplanted to agarose-coated (1% w/v in PBS) 96-well plates separately and further incubated for 48 h to mature.

***In vitro* cytotoxicity against cell lines**

The *in vitro* cytotoxicity of DOX, PEG-PCL/DOX, OPDMA-PCL/DOX, and

OPDEA-PCL/DOX was studied using the 3-(4,5-dimethyl-2-thiazolyl)-2,5-diphenyl-2-H-tetrazolium bromide (MTT) assay. 4T1, HepG2, HeLa, A549, BxPC-3, and MCF-7/ADR cells were seeded into 96-well plates at a density of 4,000 cells per well and incubated overnight at 37 °C. The cells were then exposed to different formulations for 48 h. The medium was then changed with 0.2 mL of fresh medium containing 0.75 mg/mL of MTT and incubated for another 5 h. The medium was carefully removed, and 0.1 mL DMSO was added to dissolve the dark blue formazan crystals. The absorbance (OD) in each well was measured at 562 nm and 620 nm using a multiwell plate reader. Each drug concentration was tested in triplicate in three independent experiments. Cell viability was calculated according to the following formula.

$$\text{Cell viability (\%)} = \frac{OD_{sample，562nm}\text{-}OD_{sample，620nm}}{OD_{control，562nm}\text{-}OD_{control，620nm}} \times 100\%$$

The *in vitro* cytotoxicity against MTSs

The in vitro cytotoxicity of each formulation against MTSs was evaluated by the resazurin method. Matured MTSs of MCF-7/ADR cells were established as described above. DOX, PEG-PCL/DOX, OPDMA-PCL/DOX, and OPDEA-PCL/DOX were added into each MTS-containing well at serial concentrations and incubated for 48 h. The medium was then changed with 0.2 mL of fresh medium containing 0.1 mg/mL of resazurin and incubated for another 4 h. After incubation, 100 µL medium in each well was transferred into another 96-well plate, and the fluorescence intensity (FI) in each well was measured at 590 nm (excitation 530 nm) with a microplate spectrophotometer. Each drug concentration was tested in triplicate in three independent experiments. Cell viability was calculated according to the following formula.

$$\text{Cell viability (\%)} = \frac{FI_{sample} - FI_{blank}}{FI_{control} - FI_{blank}} \times 100\%$$

Cellular uptake and subcellular distribution of Cy5-labelled micelles

MCF-7/ADR cells in 1 mL of medium were plated in glass-bottom Petri dishes at a density of 1×10^5 cells per dish and incubated for 18 h. The medium was replaced with 1 mL of fresh medium containing PEG-^{Cy5}PCL, OPDMA-^{Cy5}PCL, or OPDEA-^{Cy5}PCL at a Cy5 dose of 0.5 µg/mL. After timed incubation, the cells were further incubated with LysoTracker Green (Invitrogen, 200 nM) for 1.5 h or MitoTracker Green

(Invitrogen, 200 nM) for 0.5 h. The nuclei were stained with Hoechst33342 (Invitrogen, two drops) for 15 min. The medium was then removed, and the cells were washed thrice with cold PBS for confocal imaging (Nikon-A1 system; Tokyo, Japan). The excitation and emission wavelengths of each dye were set up following the manufacturer's instructions. Hoechst 33342 was shown in blue, LysoTracker Green and MitoTracker Green in green, and Cy5 in red. The Manders' correlation coefficient between micelles and lysosomes/mitochondria were analyzed by ImageJ.

The cellular uptake of Cy5-labelled micelles was further quantified by flow cytometry. MCF-7/ADR cells in 1 mL medium were seeded in 12-well plates at a density of 1×10^5 cells per well and incubated for 18 h. PEG-^{Cy5}PCL, OPDMA-^{Cy5}PCL, or OPDEA-^{Cy5}PCL micelle solutions were added at a final Cy5 dose of 0.5 μg/mL, followed by 0.5 or 1 h incubation. The cells were then trypsinized, washed, and resuspended in PBS (containing 0.5 mg/mL heparin) for immediate flow cytometry analysis. Cy5-positive cell ratio was then calculated.

Endocytosis inhibitor effects on cellular uptake

MCF-7/ADR cells in 1 mL medium were seeded in 24-well plates at a density of 2×10^5 cells per well and incubated for 18 h. The medium was then replaced with 1 mL serum-free medium. Filipin (an inhibitor of caveolae-mediated endocytosis, 7.5 μM), wortmannin (an inhibitor of phosphatidylinositol 3-kinases-mediated macropinocytosis, 1 μM), chlorpromazine (an inhibitor of clathrin-mediated endocytosis, 50 μM), or cytochalasin D (an inhibitor of actin-polymerization, 5 μM) was added to the medium. After 30 min incubation, PEG-^{Cy5}PCL, OPDMA-^{Cy5}PCL, or OPDEA-^{Cy5}PCL micelle solutions were added at a Cy5 dose of 0.5 μg/mL and incubated for 1 h. The cells were then trypsinized, washed, and resuspended in PBS (containing 0.5 mg/mL heparin) for immediate flow cytometry analysis.

The JC-1 assay of mitochondrial membrane potential

For JC-1 aggregates measurement, MCF-7/ADR cells in 1 mL medium were seeded in glass-bottom Petri dishes at a density of 1×10^5 cells per dish and incubated for 18 h. The medium was replaced by 1 mL of fresh medium containing DOX, PEG-PCL/DOX, OPDMA-PCL/DOX, or OPDEA-PCL/DOX at a DOX-equivalent

dose of 0.5 μg/mL or CCCP (1 mM). After 1 h of incubation, the supernatant was replaced by fresh medium, and the cells were stained with JC-1 reagent (5 μg/mL) for 20 min at 37 °C. Subsequently, the cells were washed with PBS and imaged using confocal microscopy for JC-1 aggregates visualization (excitation: 543 nm, emission: 570~620 nm).

For monomeric JC-1 measurement, MCF-7/ADR cells in 1 mL medium were seeded in 24-well plates at a density of 2 × 10^5 cells per well and incubated for 18 h. The cells were incubated with DOX, PEG-PCL/DOX, OPDMA-PCL/DOX, or OPDEA-PCL/DOX at a DOX-equivalent dose of 0.5 μg/mL or CCCP (1 mM) for 1 h. The medium was removed, and the cells were trypsinized, washed with PBS, resuspended in 300 μL of fresh medium. JC-1 reagent was added into the cells at a concentration of 5 μg/mL and incubated for 20 min at 37 °C. Then the cells were isolated and resuspended in 500 μL PBS (containing 0.5 mg/mL heparin) for flow cytometry analysis.

For determination of the ratio of JC-1 aggregates to monomers, MCF-7/ADR cells were seeded in 12-well plates at a density of 2 × 10^5 cells per well and incubated for 18 h. The cells were incubated with DOX, PEG-PCL/DOX, OPDMA-PCL/DOX, or OPDEA-PCL/DOX at a DOX-equivalent dose of 0.5 μg/mL or CCCP (1 mM) for 3 h. The medium was removed and the cells were trypsinized, washed with PBS, resuspended in 200 μL PBS. The fluorescence intensity of JC-1 monomers (Ex : 490 nm, Em : 530 nm) and aggregates (Ex : 525 nm, Em : 590 nm)in each sample was measured using a microplate spectrophotometer.

Intracellular ATP detection

MCF-7/ADR cells were seeded in 12-well plates at a density of 2 × 10^5 cells per well and incubated for 18 h. The cells were incubated with DOX, PEG-PCL/DOX, OPDMA-PCL/DOX, or OPDEA-PCL/DOX at a DOX-equivalent dose of 0.5 μg/mL or CCCP (1 mM) for 12 h. The cells were then trypsinized, lysed and centrifuged. The supernatants were collected and the intracellular ATP concentration was measured by ATP detection kit.

Exocytosis kinetics study of Cy5-labelled micelles

ECDHCC-1 cells in 1 mL medium were seeded in 24-well plates at a density of 2 × 10^5 cells per well and incubated for 18 h. PEG-^{Cy5}PCL, OPDMA-^{Cy5}PCL, or OPDEA-^{Cy5}PCL micelle solutions were added into the medium at a Cy5-equivalent dose of 0.5 µg/mL. After 3 h, the cells were re-cultured for timed intervals and then trypsinized, washed and resuspended in PBS (containing 0.5 mg/mL heparin) for immediate flow cytometry analysis.

Transepithelial study of Cy5-labelled micelles across the ECDHCC-1 cell monolayer

ECDHCC-1 cells in 200 µL medium were seeded onto transwell polycarbonate cell culture inserts at a density of 2 × 10^5 cells per well, while 1 mL medium was added into the basolateral compartment. When the trans-endothelial electrical resistance (TEER) value determined by epithelial volt/ohm meter (Millicell ERS-2, Millipore) reached 500 $\Omega \cdot cm^2$, the medium was replaced by 200 µL fresh medium containing PEG-^{Cy5}PCL, OPDMA-^{Cy5}PCL, or OPDEA-^{Cy5}PCL micelles at a Cy5-equivalent dose of 0.5 µg/mL. At timed intervals, 200 µL of the medium in the basolateral compartment were collected, and micelle concentrations were measured by detecting Cy5 fluorescence intensity with a spectrophotometer.

For the effects of endo/exocytosis inhibitors, cytochalasin D (5 µM), monensin (50 µM), brefeldin A (90 µM) or nocodazole (30 µM) was added into both apical and basolateral side. After 3 h, PEG-^{Cy5}PCL, OPDMA-^{Cy5}PCL, or OPDEA-^{Cy5}PCL micelles were added into the apical side at a Cy5-equivalent dose of 0.5 µg/mL. After 24 h, 200 µL of the medium in the basolateral compartment were collected, and micelle concentrations were measured by detecting Cy5 fluorescence intensity with a spectrophotometer. Temperature-dependent measurements were performed at 4 °C.

For simulation of cellular uptake of the transported micelles, coverslips of MCF-7/ADR cells were set on the basolateral side. After 24 h transportation of PEG-^{Cy5}PCL, OPDMA-^{Cy5}PCL, or OPDEA-^{Cy5}PCL micelle across the ECDHCC-1 cell monolayer at a Cy5-equivalent dose of 0.5 µg/mL, MCF-7/ADR cells on the coverslips were both visualized using confocal microscopy and measured using a flow cytometry.

Intercellular transport of Cy5-labeled micelles between MCF-7/ADR cells

MCF-7/ADR cells in 1 mL medium were seeded in glass-bottom Petri dishes at a density of 1×10^5 cells per dish and incubated for 18 h. The cells (1st batch) were cultured with PEG-^{Cy5}PCL, OPDMA-^{Cy5}PCL, or OPDEA-^{Cy5}PCL micelle at a Cy5-equivalent dose of 1 μg mL^{-1} for 6 h, washed with PBS, and imaged with confocal microscopy. The cells were then cultured in 0.8 mL of fresh medium for 12 h, and the medium was harvested to incubate the 2nd batch of cells for 12 h, followed by washing with PBS (containing 0.5 mg/mL heparin) and imaging with confocal microscopy. The same procedures were implemented for another two rounds.

Penetration of Cy5-labelled micelles in MCF-7/ADR MTSs

Matured MTSs of MCF-7/ADR cells in 96-well plates were incubated with PEG-^{Cy5}PCL, OPDMA-^{Cy5}PCL, or OPDEA-^{Cy5}PCL micelle at a Cy5-equivalent dose of 0.5 μg/mL for 4 h. Then the MTSs were washed with PBS (containing 0.5 mg/mL heparin), transferred to ibidi μ-Slide 8 well polymer coverslips, and imaged using a confocal microscope.

To evaluate the effect of endo/exocytosis inhibitors, the MTSs were pretreated with cytochalasin D (5 μM) or brefeldin A (90 μM) for 10 h and then incubated with PEG-^{Cy5}PCL, OPDMA-^{Cy5}PCL, or OPDEA-^{Cy5}PCL micelles at a Cy5-equivalent dose of 0.5 μg/mL for 4 h. Then the MTSs were washed with PBS (containing 0.5 mg/mL heparin), transferred to a chambered coverslip, and imaged using a confocal microscope.

Penetration of DOX-loaded micelles in MCF-7/ADR MTSs

Matured MTSs of MCF-7/ADR cells were incubated in 96-well plates with DOX, PEG-PCL/DOX, OPDMA-PCL/DOX, or OPDEA-PCL/DOX at a DOX concentration of 1 μg/mL. After 4 h incubation, the MTSs were washed with PBS containing 0.5 mg/mL heparin, transferred to a chambered coverslip, and imaged using a confocal microscope. The distribution of DOX fluorescence was analyzed using ImageJ.

Mice

Mice were provided by the Institute of Medicine of Zhejiang Province and maintained in a specific pathogen-free (SPF), temperature-controlled (22 ^{o}C ± 1 ^{o}C)

animal facility on a reverse 12-hour light, 12-hour dark cycle at Zhejiang University. Food and water were given ad libitum. Mice were handled under the protocols approved by the Institutional Animal Care and Use Committee (IACUC) of Zhejiang University (approval number: 20379) in accordance with the institutional guidelines.

Adsorption of micelles on red blood cells (RBCs)

Blood samples of female ICR mice (6-8 weeks old) were collected into EDTA-rinsed tubes and centrifuged at 300 g for 5 min to remove the plasma. RBCs were isolated by washing with erythrocyte buffer (containing 150 mM NaCl and 6 mM glucose in DI water) and centrifugation (300 g for 5 min) for three cycles and finally suspended in erythrocyte buffer to obtain the RBC suspension (2×10^6 cells/mL). To 1 mL of the RBC suspensions were added PEG-PCL/DOX, OPDMA-PCL/DOX, or OPDEA-PCL/DOX (DOX-eq. 1 µg/mL) and incubated at 37 °C for 15 min in the dark. FITC-labeled wheat germ agglutinin (^{FITC}WGA, 1 µg/mL) was added and incubated for another 15 min. RBCs were isolated by centrifugation and observed using a confocal microscope.

***In vivo* pharmacokinetic study**

Female ICR mice (6-8 weeks old) were randomly grouped (n = 3): (1) DOX, (2) PEG-PCL/DOX, (3) OPDMA-PCL/DOX, and (4) OPDEA-PCL/DOX. Each formulation was *i.v.* injected via the tail vein at a DOX-eq dose of 4 mg/kg. At timed intervals (2 min, 0.5, 1, 2, 4, 8, 12, and 24 h), blood samples (100 µL) were drawn via the orbit venous plexus and centrifuged at 6000 rpm for 6 min at 4°C. The supernatant (50 µL) was diluted with 50 µL PBS, and the DOX concentration was determined by measuring UV absorbance at 480 nm.

Determination of DOX distribution

Female BALB/c nude mice (6–8 weeks) were subcutaneously inoculated with MCF-7/ADR cells (5×10^7 cells/mL, 200 µL) at the right flank. When the tumors reached ~150 mm^3, the mice were randomized into three groups (n = 3 per group) and *i.v.* injected with 200 µL of DOX, PEG-PCL/DOX, OPDMA-PCL/DOX, or OPDEA-PCL/DOX micelles (DOX-eq. dose 4 mg/kg). The mice were sacrificed at 24 h post-treatment, and tumors and major organs (heart, liver, spleen, lung, and kidneys)

were excised. The organs or tissues were homogenized, and DOX was extracted with acetonitrile containing 0.5% acetic acid. The mixture was centrifuged at 13,000 rpm for 10 min, and the supernatant was concentrated and subjected to HPLC to determine DOX contents.

***In vivo* real-time distribution**

Female BALB/c nude mice (6–8 weeks) were subcutaneously inoculated with MCF-7/ADR cells (5 × 10^7 cells/mL, 200 µL) at the right flank. When the tumors reached ~150 mm^3, the mice were randomized into three groups (n = 3 per group) and *i.v*. injected with 200 µL of PEG-^{Cy5}PCL, OPDMA-^{Cy5}PCL, and OPDEA-^{Cy5}PCL micelles (Cy5-eq. dose 0.5 mg/kg). At 0.5, 2, 6, 12, 24, 36, and 48 h post-injection, mice were anesthetized with isoflurane and imaged using a Caliper IVIS Lumina II imaging system (Pekin Elemer, USA). After the final imaging, mice were sacrificed, and tumors and major organs (heart, liver. spleen, lung, and kidney) were collected for *ex vivo* imaging. Exposure time was 2 s per image, and the depth was 1.5 cm. The fluorescence intensity in each tumor and organ was quantified using the IVIS Spectrum Software.

***In vivo* extravasation from blood vessels and tumor penetration of micelles**

The real-time visualization of blood vessel extravasation and tumor penetration of micelles was performed as previously reported.[2] MCF-7/ADR cells (5 × 10^7 cells/mL, 200 µL) were *s.c.* injected at the lower right abdomen near a large vein in mice. When the tumor volume reached ~40 mm^3, mice were anesthetized with 1% sodium pentobarbital, and the skin was scissored subcutaneously along the middle line of the abdomen to expose the tumor without breaking the surrounding veins. Then the tumor was moistened with glycerol and fixed onto a microscope slide using histoacryl. After a single *i.v.* injection of 200 µL of PEG-^{Cy5}PCL, OPDMA-^{Cy5}PCL, or OPDEA-^{Cy5}PCL micelles (Cy5.5-eq. dose 0.5 mg/kg), the tumor region with large veins and microvessels was imaged at timed intervals using a confocal microscope. The fluorescence density as a function of distance from blood vessels was analyzed by ImageJ.

***In vivo* anti-tumor efficacy**

Female BALB/c nude mice (6-8 weeks old) bearing MCF-7/ADR tumors of ~80 mm^3 were randomly divided into 5 groups (n=5) and *i.v.* injected with 0.2 mL of PBS, DOX, PEG-PCL/DOX, OPDMA-PCL/DOX, or OPDEA-PCL/DOX at a DOX-equivalent dose of 4 mg/kg every three days for five times. The tumor volume and body weight were measured individually on alternate days. On day 21 post-treatment, mice were euthanized, and tumors and major organs (heart, liver, spleen, lung, kidney) were collected. The tumor inhibition rate (TIR) was calculated following the equation: TIR = (mean tumor weight of the control group − mean tumor weight of the treatment group)/mean tumor weight of control group×100%.

Histological analysis

Tumor and organ samples were fixed with 4% paraformaldehyde in PBS and embedded in paraffin. Tissue sections were sliced (5 μm thick) and stained with hematoxylin-eosin (H&E, Beyotime, China) for microscopy examination.

Statistical analysis

Statistical analysis was performed using GraphPad Prism and Excel. The two-tailed, unpaired Students' t-test was utilized to calculate the significance of two populations. Data were presented as mean ± SD. $P < 0.05$ was regarded as statistically significant.

Supplementary figures

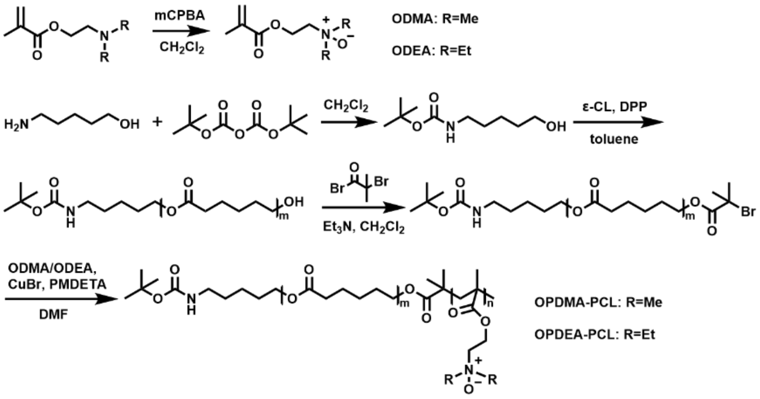

Scheme S1. Synthetic routes to OPDMA-PCL and OPDEA-PCL.

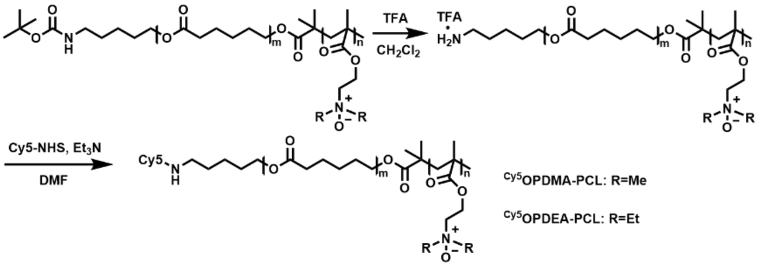

Scheme S2. Synthetic routes to fluorescently labeled OPDMA-^{Cy5}PCL and OPDEA-^{Cy5}PCL.

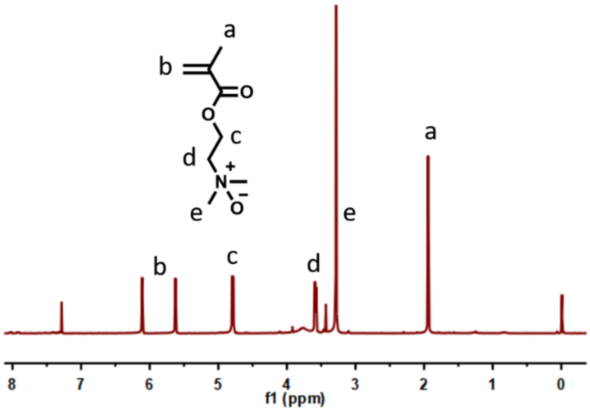

Figure S1. ^{1}H-NMR spectrum of ODMA in $CDCl_3$.

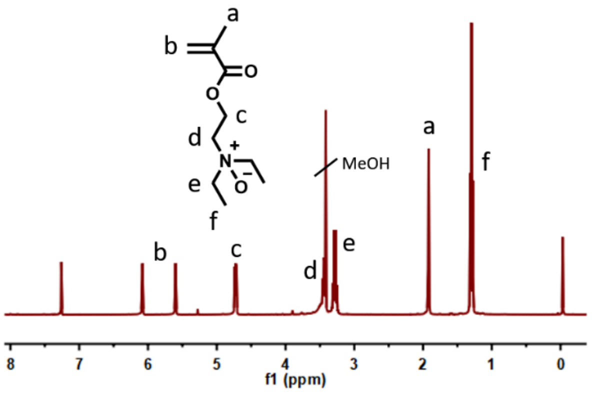

Figure S2. ^{1}H-NMR spectrum of ODEA in $CDCl_3$.

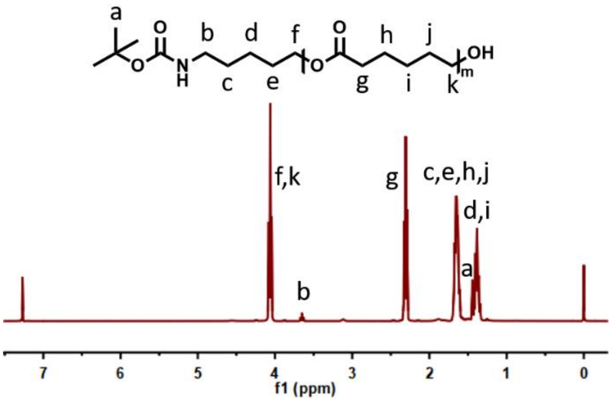

Figure S3. ^{1}H-NMR spectrum of BocNH-PCL-OH in $CDCl_3$.

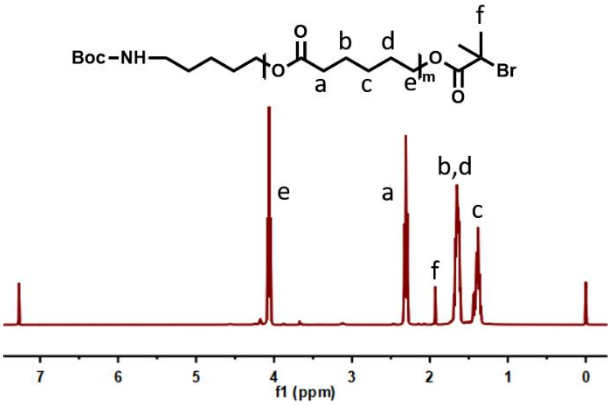

Figure S4. ^{1}H-NMR spectrum of PCL-Br in $CDCl_3$.

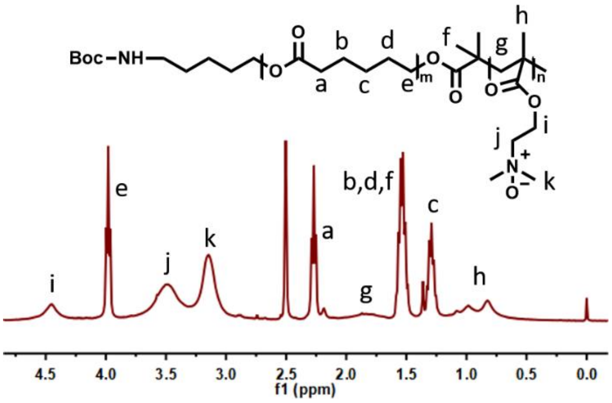

Figure S5. ^{1}H-NMR spectrum of OPDMA-PCL in DMSO-d6.

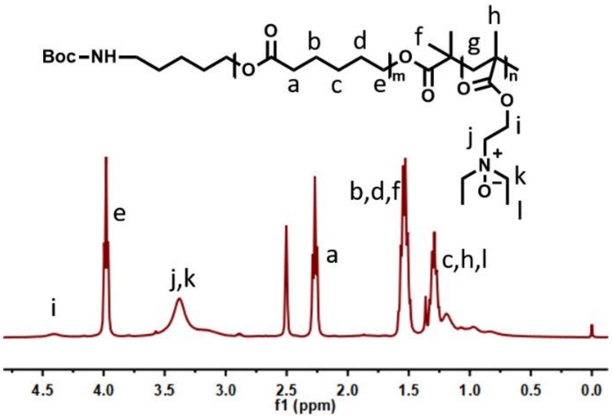

Figure S6. ^{1}H-NMR spectrum of OPDEA-PCL in DMSO-d6.

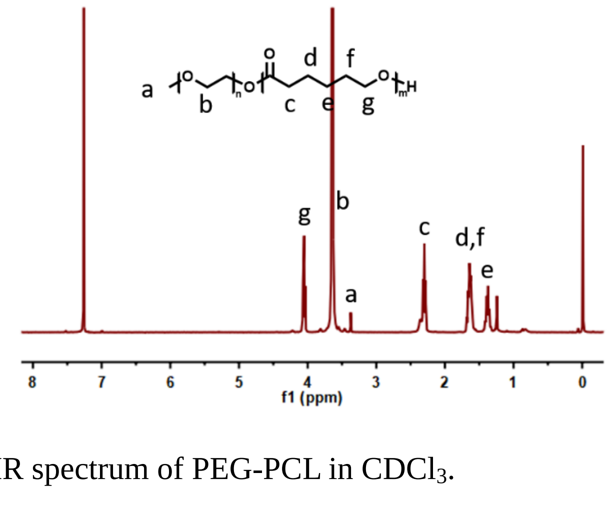

Figure S7. ^{1}H-NMR spectrum of PEG-PCL in $CDCl_3$.

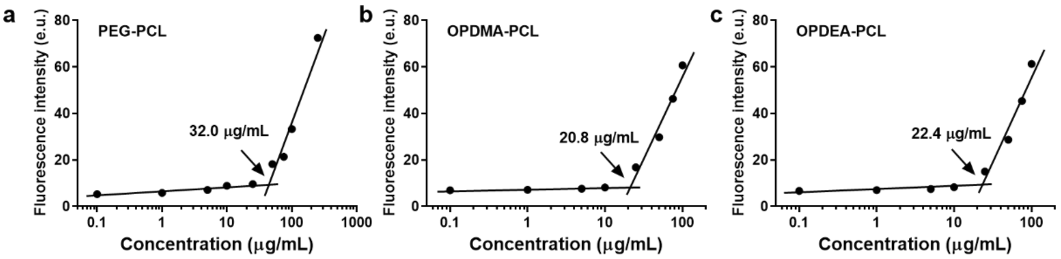

Figure S8. CMCs of **a)** PEG-PCL, **b)** OPDMA-PCL, and **c)** OPDEA-PCL as determined by the Nile red method.

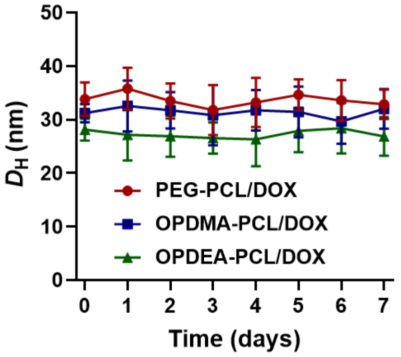

Figure S9. The stability of PEG-PCL/DOX, OPDMA-PCL/DOX, and OPDEA-PCL/DOX in PBS (10 mM, pH 7.4).

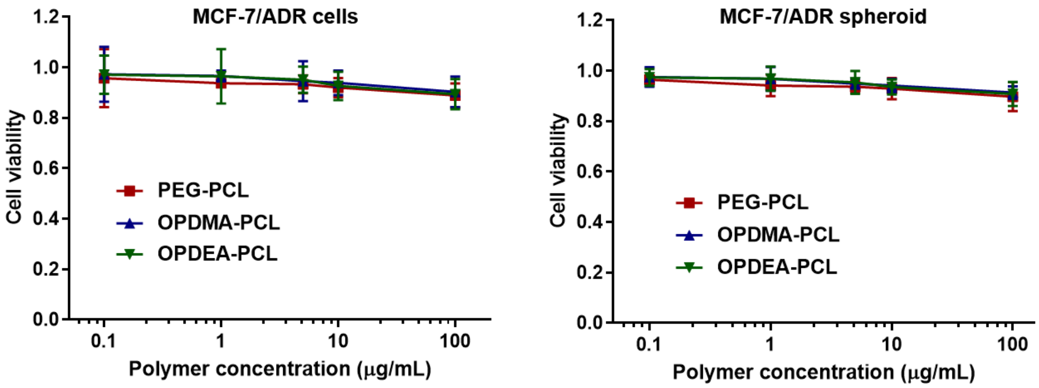

Figure S10. The *in vitro* cytotoxicity of PEG-PCL, OPDMA-PCL, OPDEA-PCL micelles against various cancer cell lines (48 h treatment).

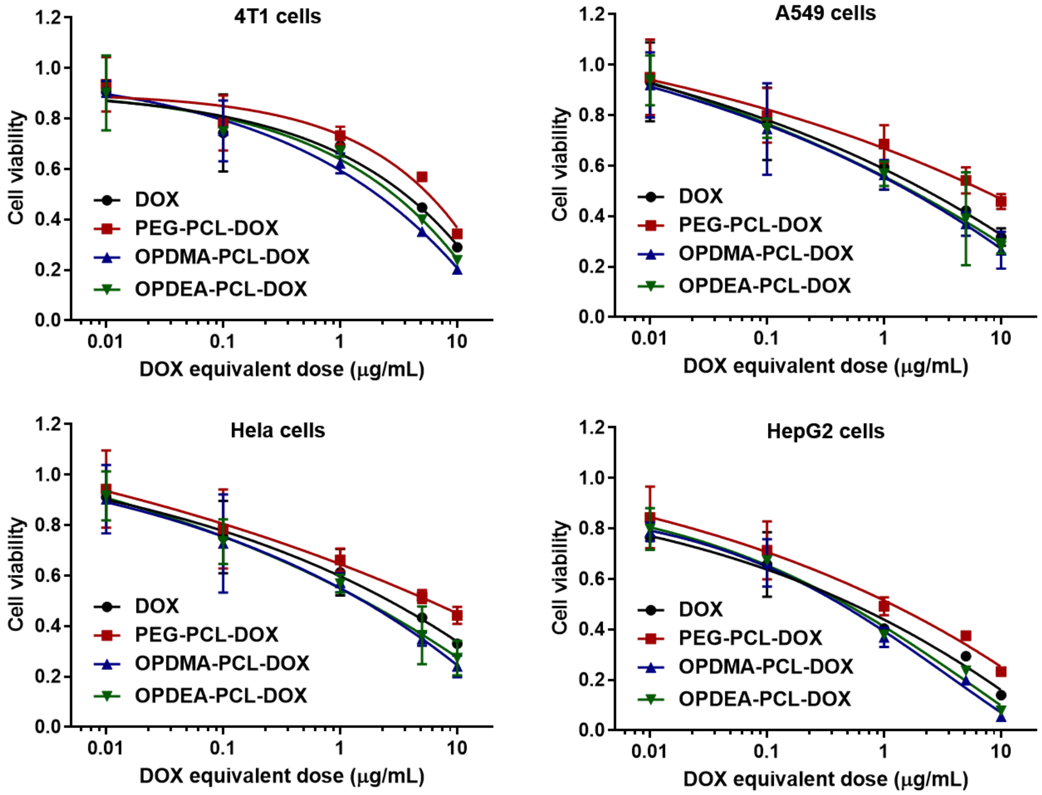

Figure S11. The *in vitro* cytotoxicity of DOX, PEG-PCL/DOX, OPDMA-PCL/DOX, OPDEA-PCL/DOX micelles against various cancer cell lines (48 h treatment).

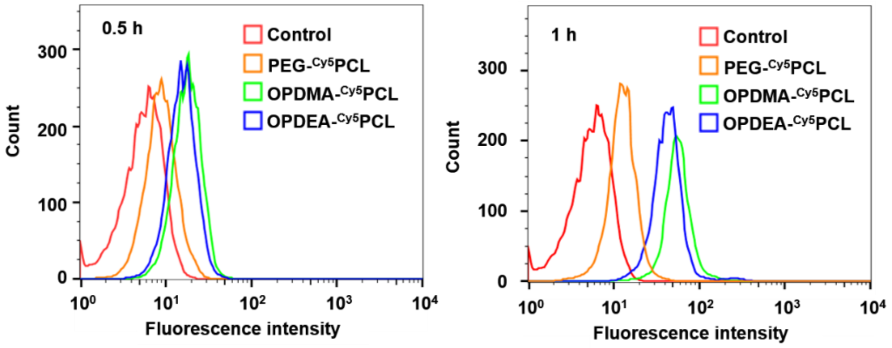

Figure S12. Uptake of PEG-^{Cy5}PCL, OPDMA-^{Cy5}PCL, and OPDEA-^{Cy5}PCL micelles by MCF-7/ADR cells measured by flow cytometry. Cy5-eq. dose: 0.5 μg/mL.

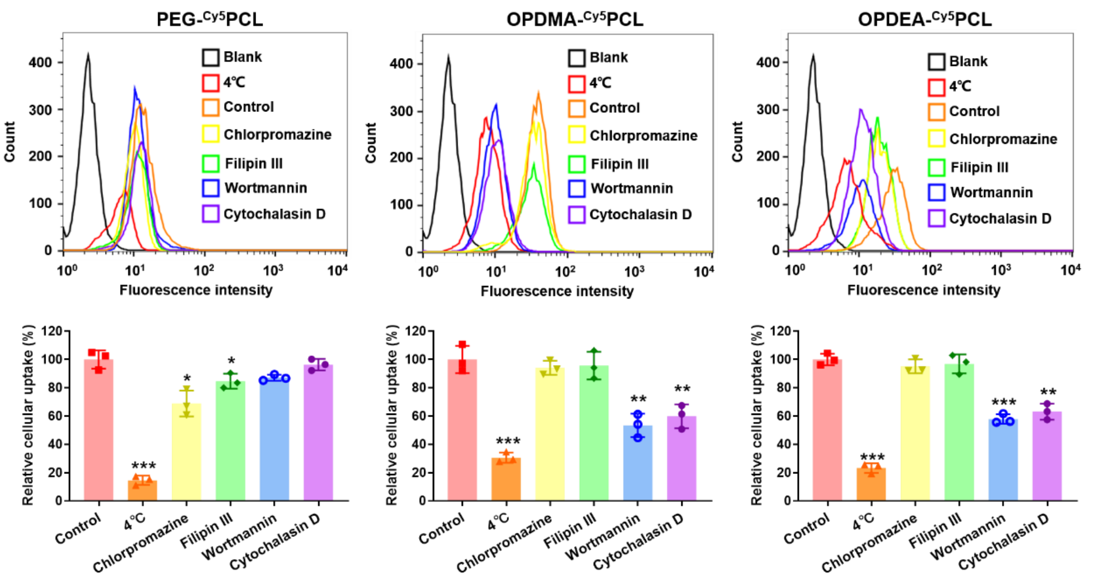

Figure S13. Effects of inhibitors on cellular uptake of the fluorescently labeled micelles in MCF-7/ADR cells. Cells were treated with inhibitors or incubated at 4 °C for 0.5 h, and then incubated with PEG-^{Cy5}PCL, OPDMA-^{Cy5}PCL, or OPDEA-^{Cy5}PCL for 1 h (Cy5-eq. 0.5 μg/mL). After wash with PBS, the cells were isolated and analyzed using flow cytometry.

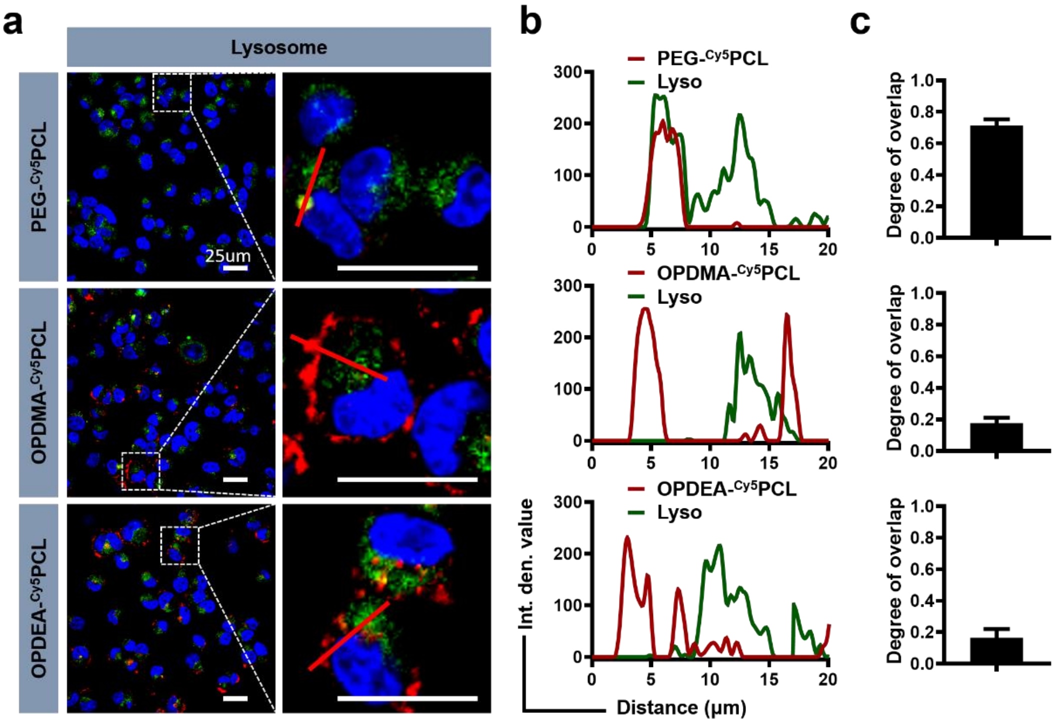

Figure S14. a) Colocalization of PEG-^{Cy5}PCL, OPDMA-^{Cy5}PCL, and OPDEA-^{Cy5}PCL with lysosome after 2-hour incubation. **b)** Overlapping profiles of

Cy5 fluorescence with LysoTracker Green fluorescence along the selected red line across the cell. **c)** Manders' correlation coefficients of Cy5 and LysoTracker Green calculated by pixel intensity using ImageJ. Cells were cultured with PEG-^{Cy5}PCL, OPDMA-^{Cy5}PCL, and OPDEA-^{Cy5}PCL (Cy5-eq. dose: 0.1 μg/mL) for 2 h. The Cy5 fluorescence was shown in red and the LysoTracker Green in green. Scale bar: 25 μm.

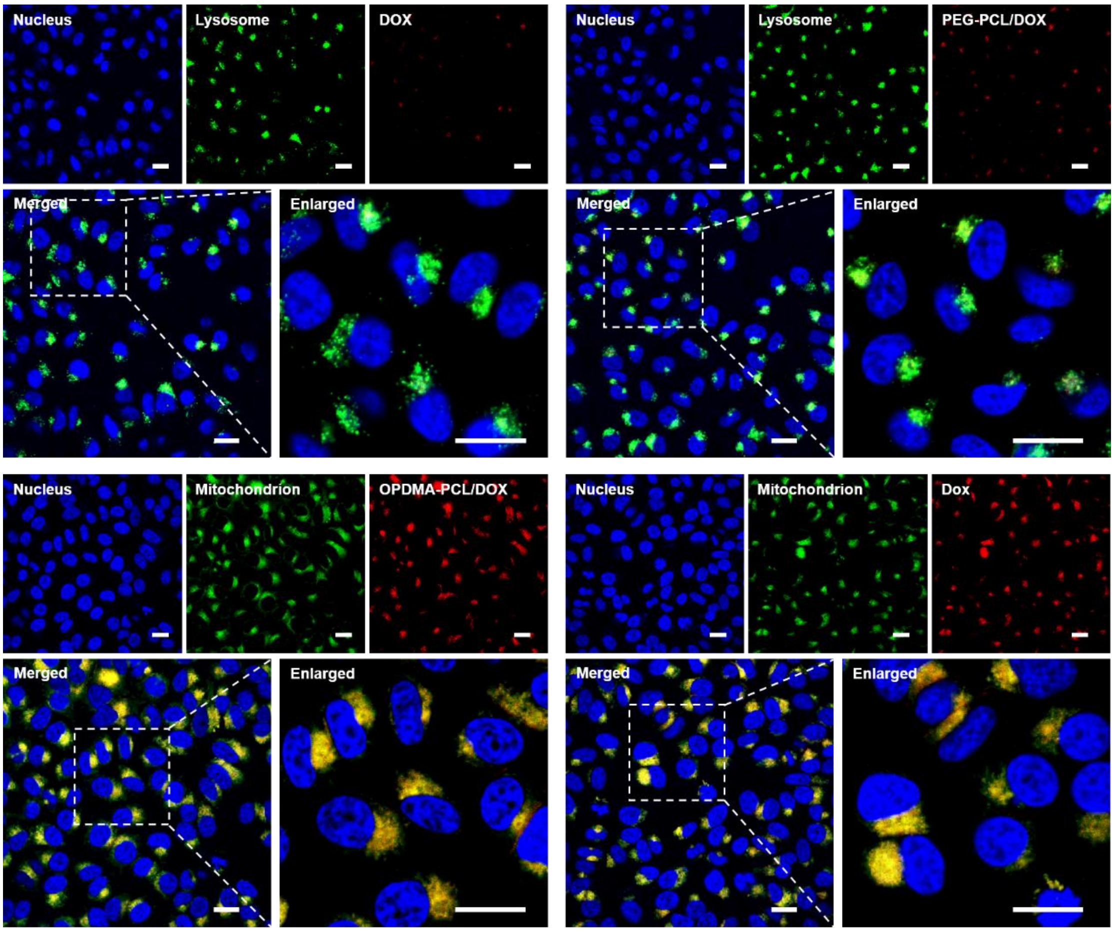

Figure S15. The subcellular distribution of DOX-loaded micelles in MCF-7/ADR cells. Cells were incubated with PEG-PCL/DOX, OPDMA-PCL/DOX or OPDEA-PCL/DOX for 2 h (DOX-eq. 5 μg/mL). After wash with PBS, the cells were imaged by a confocal microscope. Lysosomes were labeled with LysoTracker Green, and mitochondria were labeled with MitoTracker Green. Scale bar: 20 μm.

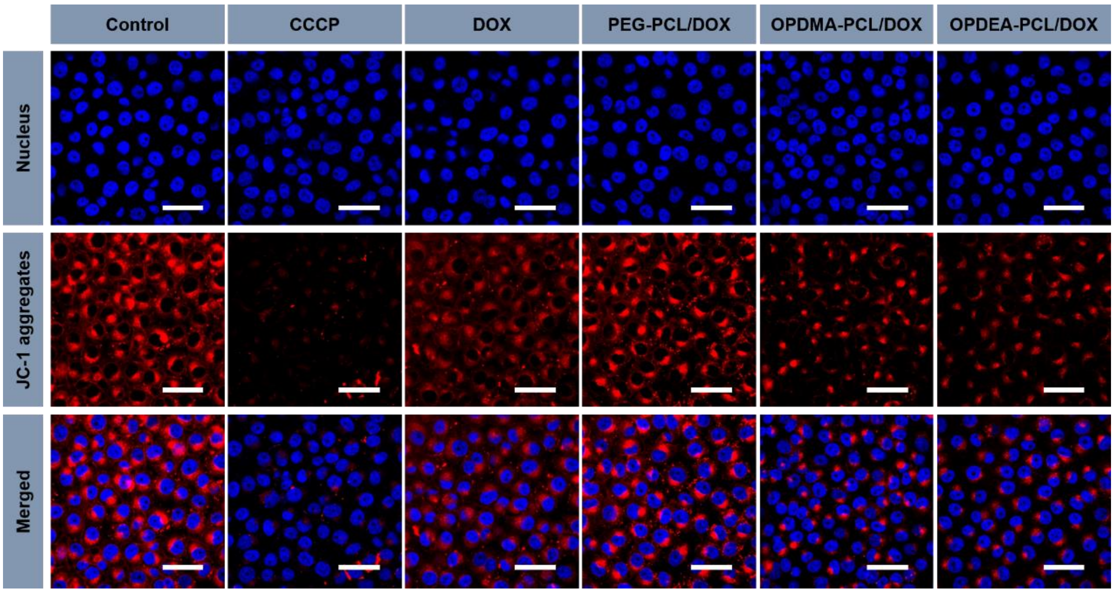

Figure S16. Confocal microscopy images of MCF-7/ADR cells stained with the JC-1 dye. The JC-1 aggregate is shown in red. Scale bar: 50 μm. The cells were incubated with DOX formulations at a DOX-eq. dose of 0.5 μg/mL or CCCP (1 mM) for 1 h.

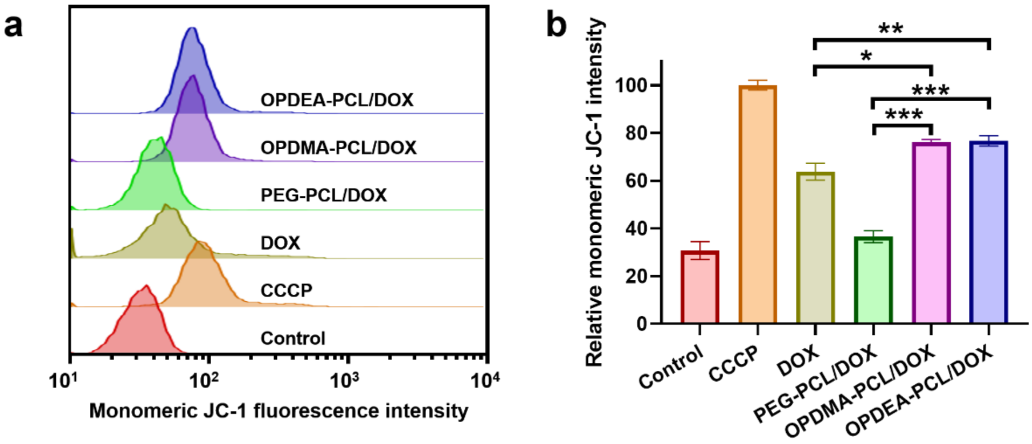

Figure S17. JC-1 assay of the $\Delta\Psi_m$ in MCF-7/ADR cells. **a)** Flow cytometry analysis and **b)** quantification of the monomeric JC-1. The cells were incubated with DOX formulations at a DOX-eq. dose of 0.5 μg/mL or CCCP (1 mM) for 1 h.

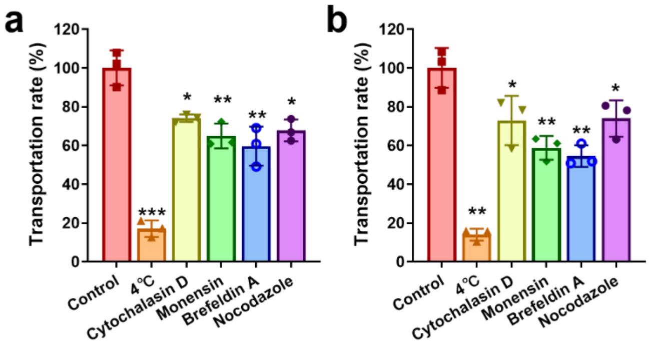

Figure S18. The relative transportation rates of **a)** OPDMA-^{Cy5}PCL and **b)** OPDEA-^{Cy5}PCL micelles in the presence of transcytosis inhibitors. The media in both sides were added with inhibitors. After 24 h, the micelle concentration in the basolateral compartment was measured.

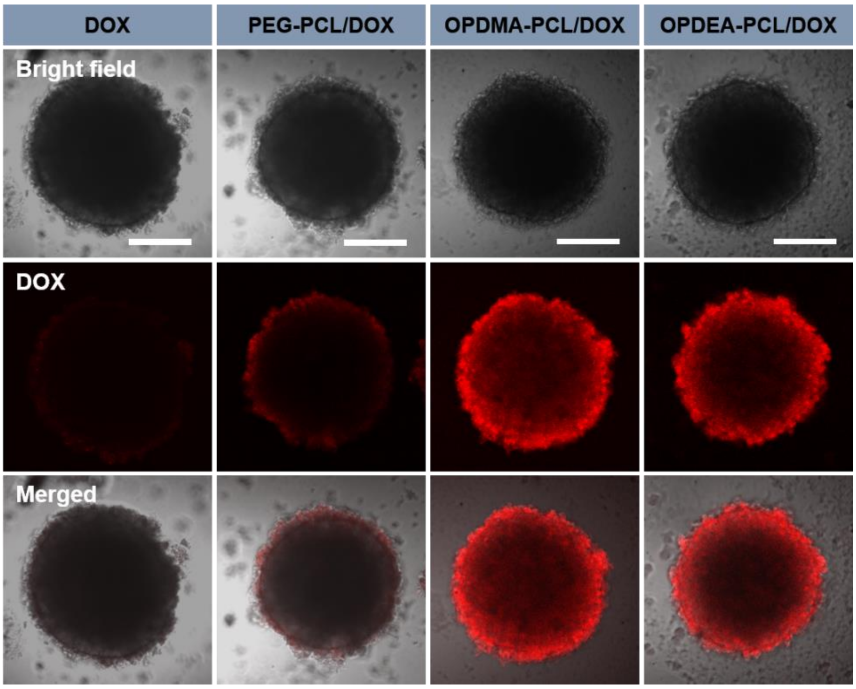

Figure S19. *In vitro* penetration of DOX-loaded micelles in MCF-7/ADR MTSs. The MTSs were exposed to different DOX formulations for 4 h (DOX-eq. dose of 5 μg/mL) and then washed twice with 10% heparin-containing PBS, and the images were acquired at the middle layers using a confocal microscope. Scale bar: 250 μm.

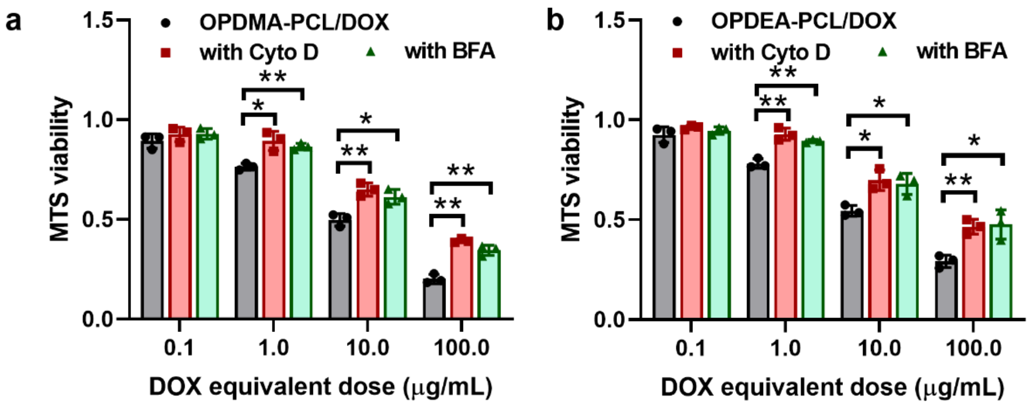

Figure S20. The effects of endo/exocytosis inhibitors on the cytotoxicity of **a)** OPDMA-PCL/DOX and **b)** OPDEA-PCL/DOX against the MCF-7/ADR MTSs. MTSs were pretreated with cytochalasin D (10 μM) or brefeldin A (10 μM) for 10 h and then incubated with OPDMA-PCL/DOX or OPDEA-PCL/DOX for 48 h. The cytotoxicity was determined by the resazurin assay. *P < 0.05, **P < 0.01.

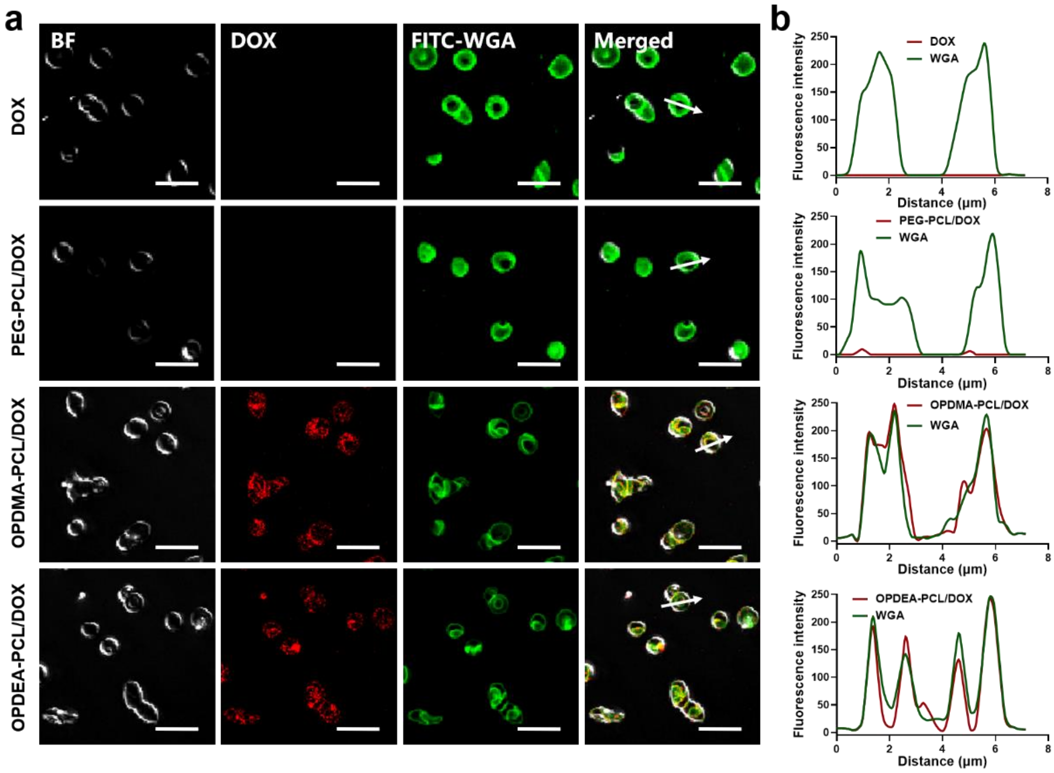

Figure S21 Adherence of DOX, PEG-PCL/DOX, OPDMA-PCL/DOX, and OPDEA-PCL/DOX to RBCs. a) Confocal images of RBCs after 0.5 h incubation with each formulation at DOX-equivalent dose of 1 μg/mL. Scale bar: 10 μm. **b)**

Colocalization of ^{FITC}WGA-stained RBC membrane and DOX or DOX-loaded micelles along the selected vectors (white arrow) in confocal images of a randomly selected RBC.

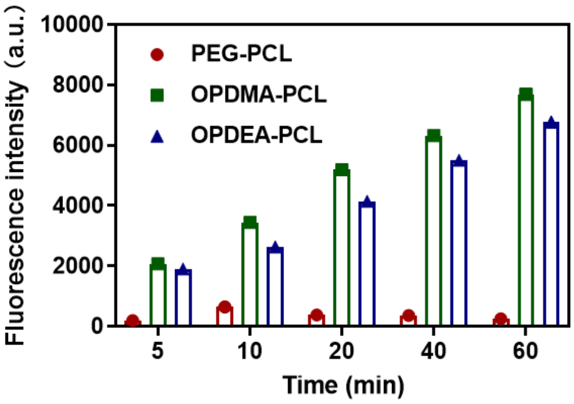

Figure S22. Cy5 integrated fluorescence intensity of each micelle along the selected line in Figure 6f-h at different times.

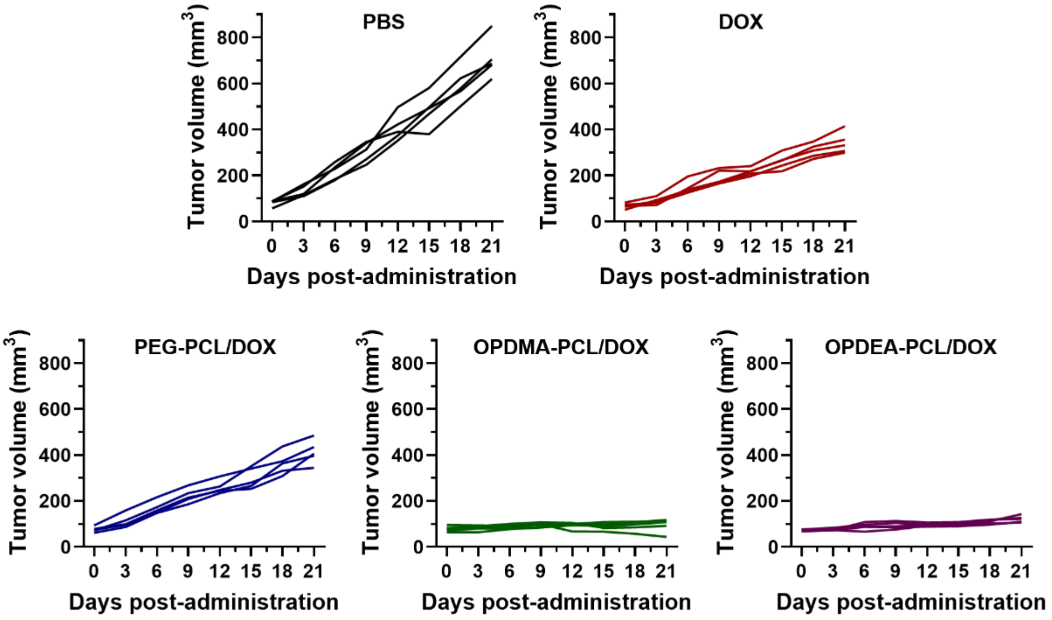

Figure S23. Tumor growth curves of the individual mouse in each group.

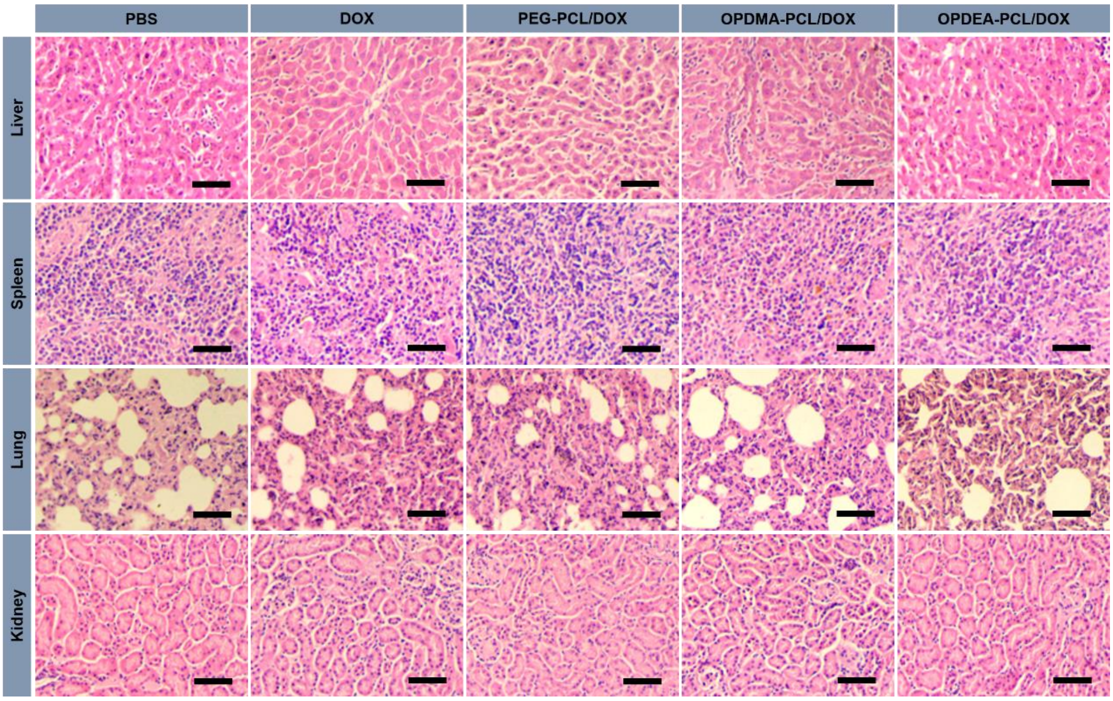

Figure S24. Representative histological features of the main organs. The tissue sections were stained with hematoxylin-eosin and examined by a light microscope. Scale bar: 50 μm.

Table S1. Characterizations of different kinds of polymeric micelles

Polymers	PEG-PCL	OPDMA-PCL	OPDEA-PCL
$M_{n,\mathrm{GPC}}$	30000	28000	29000
$M_{w,\mathrm{GPC}}$	32000	30000	31000
PDI[a]	1.07	1.07	1.08
CMC (µg/mL)[c]	32.0	20.8	22.4
D_H (nm)[b, *]	31.2±1.3	29.4±1.7	24.8±1.8
PDI [b, *]	0.121±0.037	0.108±0.052	0.102±0.071
Zeta potential (mV)[b, *]	-2.85±0.48	-2.08±0.92	-3.46±0.73
D_H (nm)[b, **]	35.2±1.7	32.1±2.3	26.4±1.6
PDI [b, **]	0.136±0.074	0.124±0.031	0.118±0.059
Zeta potential (mV)[b, **]	-3.13±0.85	-2.62±0.74	-4.08±0.83
D_H (nm)[b, ***]	37.4±1.3	34.5±1.4	27.7±1.1
PDI [b, ***]	0.189±0.034	0.176±0.042	0.168±0.038
Zeta potential (mV)[b, ***]	-8.60±0.18	-6.07±0.78	-9.15±0.29
Drug loading efficiency (%)[d]	85.4	82.3	80.8
Drug loading content (%)[d]	16.3	15.7	14.9

a, Polydispersity index (PDI) determined by GPC. *b*, PDI determined by DLS. *c*, Critical micelle concentration (CMC) determined by a Nile red fluorescent method. *d*, Encapsulation efficiency and loading efficiency of DOX calculated by the mass of DOX packing and corresponding batch charge. *, blank micelles. **, Cy5-labeled blank micelles. ***, DOX-loaded micelles.

Table S2. IC50 values of DOX, PEG-PCL/DOX, OPDMA-PCL/DOX and OPDEA-PCL/DOX against various cell lines and their tumor spheroids, 48 h treatments (Units: μg/mL).

Cell lines	DOX	PEG-PCL/ DOX	OPDMA-PCL/ DOX	OPDEA-PCL/ DOX
4T1	2.39	4.96	1.22	1.63
HepG2	0.30	0.79	0.20	0.25
HeLa	2.23	5.03	1.15	1.38
A549	2.08	6.09	1.39	1.61
BxPC-3	1.37	4.86	0.55	0.63
MCF-7/ADR	17.95	36.97	2.84	4.11
BxPC-3 MTS	> 10	> 10	1.49	2.18
MCF-7/ADR MTS	98.62	> 100	9.93	18.96

Table S3. Pharmacokinetic parameters of DOX, PEG-PCL/DOX, OPDMA-PCL/DOX, and OPDEA-PCL/DOX at a DOX-eq. dose of 4 mg/kg.

Parameter	DOX	PEG-PCL/ DOX	OPDMA-PCL/ DOX	OPDEA-PCL/ DOX
$T_{1/2}\ \alpha$ (h)	0.11±0.04	0.35±0.07	0.37±0.12	0.42±0.15
$T_{1/2}\ \beta$ (h)	0.35±0.11	5.04±1.39	6.53±1.87	5.45±1.56
$AUC_{0\sim t}$ ($\mu g \cdot mL^{-1} \cdot h^{-1}$)	5.19±3.28	100.72±22.78	109.64±31.84	71.08±17.51

References

[1] M. Zanoni, F. Piccinini, C. Arienti, A. Zamagni, S. Santi, R. Polico, A. Bevilacqua, A. Tesei, *Sci. Rep.* **2016**, *6*, 19103.

[2] S. Chen, Y. Zhong, W. Fan, J. Xiang, G. Wang, Q. Zhou, J. Wang, Y. Geng, R. Sun, Z. Zhang, Y. Piao, J. Wang, J. Zhuo, H. Cong, H. Jiang, J. Ling, Z. Li, D. Yang, X. Yao, X. Xu, Z. Zhou, J. Tang, Y. Shen, *Nat. Biomed. Eng.* **2021**, *5*, 1019.
